# Construction of a Biomimetic Tubular Scaffold Inspired by Sea Sponge Structure: Sponge‐Like Framework and Cell Guidance

**DOI:** 10.1002/advs.202416627

**Published:** 2025-02-25

**Authors:** Si Meng, Nihuan Wu, Jie Fang, Yidan Yu, Xin Tang, Yihan Wang, Xiaokang Deng, Cheng Qi, Tiantian Kong, Tengda Ding, Zhou Liu

**Affiliations:** ^1^ College of Chemistry and Environmental Engineering Shenzhen University Shenzhen Guangdong 518000 China; ^2^ Department of Biomedical Engineering School of Medicine Shenzhen University Shenzhen Guangdong 518000 China; ^3^ Guangdong Provincial Key Laboratory of Micro/Nano Optomechatronics Engineering College of Mechatronics and Control Engineering Shenzhen University Shenzhen Guangdong 518000 China; ^4^ Department of Urology Shenzhen Institute of Translational Medicine The First Affiliated Hospital of Shenzhen University Shenzhen Second People's Hospital Shenzhen Guangdong 518037 China

**Keywords:** bio‐inspired, celluloses, fiber, scaffolds, tissue engineering

## Abstract

Engineering hollow fibers with precise surface microstructures is challenging; yet, essential for guiding cells alignment and ensuring proper vascular tissue function. Inspired by *Euplectella* sponges, a novel strategy to engineer biomimetic hollow fibers with spiral surface microstructures is developed. Using oxidized bacterial cellulose, bacterial cellulose, and polydopamine, a “brick‐and‐mortar” scaffold is created through precise shear control during microfluidic coaxial spinning. The scaffold mimics natural extracellular matrices, providing mechanical stability and supporting cell growth. In vitro studies show successful co‐culture of endothelial cells (ECs) and smooth muscle cells (SMCs), with SMCs aligning along spiral surface microstructures and ECs forming a confluent inner layer. In vivo implantation confirms biocompatibility, biodegradability, and low immunogenicity. This *Euplectella*‐inspired scaffold presents a promising approach for vascular tissue engineering and regenerative medicine.

## Graphical abstract

Inspired by the structure of sea sponges, a unique method involving microfluidic coaxial spinning and in situ polymerization is designed. Biomimetic tubular scaffolds ae fabricated. Composed of specific materials such as oxidized bacterial cellulose, bacterial cellulose and polydopamine, these scaffolds have excellent mechanical stability and biocompatibility and can guide cell growth precisely, showing great promise in vascular tissue engineering.

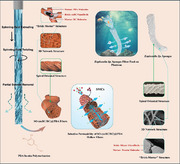

## 1 Introduction

The spatial organization of cells in tissues and organs, including their positioning and alignment, is crucial for cell behavior and tissue structure, ultimately impacting organ functionality and performance. For instance, the alignment of nerve cells is crucial for the rapid transmission of electrical signals;^[^ ^1^, ^2^, ^3^ ^]^ while, the organized arrangement of myocardial cells ensures effective heart contractions.^[^ ^4^, ^5^ ^]^ Similarly, the circumferential spiral orientation of smooth muscle cells (SMCs) around tubular organs such as blood vessels is essential for their mechanical strength and functional dynamics.^[^ ^6^, ^7^, ^8^, ^9^ ^]^

To achieve cellular organization in engineered tissue constructs, the surface microstructures, especially oriented ones, of the underlying biomaterial scaffolds play a critical role.^[^ ^10^, ^11^, ^12^, ^13^, ^14^, ^15^, ^16^ ^]^ For instance, oriented microstructures such as microfibers,^[^ ^17^, ^18^, ^19^, ^20^ ^]^ micro‐protrusions,^[^ ^21^, ^22^, ^23^ ^]^ and micro‐grooves^[^ ^24^, ^25^, ^26^ ^]^ have been shown to efficiently guide cell arrangement and orientation, facilitating cell migration and alignment. In addition, research has demonstrated that nano‐scale oriented microstructures are especially effective in guiding cell organization, with optimal results observed at scales of tens of nanometers.^[^ ^27^ ^]^ Therefore, precise construction of nano‐scale oriented microstructures on scaffolds is of great importance for controlling cellular organization in engineered tissue constructs.

Among various biomaterial scaffolds with microstructures, tubular scaffolds are particularly crucial because they mimic the structure of many vital organs, such as blood vessels, tracheae, and intestines.^[^ ^28^, ^29^, ^30^, ^31^ ^]^ These organs require precise microstructural features to support proper cell alignment, material exchange, and mechanical stability. Although various techniques, such as laser engraving,^[^ ^32^, ^33^, ^34^ ^]^ 3D printing,^[^ ^35^, ^36^, ^37^, ^38^ ^]^ and oriented electrospinning,^[^ ^39^, ^40^, ^41^, ^42^ ^]^ are available for creating oriented microstructures, each faces significant challenges when used for tubular scaffolds. For instance, laser engraving struggles with non‐planar surfaces, and typical 3D bio‐printing lacks the necessary resolution, being limited to ≈10 µm, which is significantly larger than the nano‐scale required for optimal cell induction. Oriented electrospinning, although capable of producing nano‐fiber structures, often aligns fibers axially rather than in the circular spiral direction commonly required for tubular scaffolds. Further, the requirement for thin, porous walls in tubular scaffolds introduces additional challenges in terms of maintaining structural integrity and ensuring effective cell–material interactions, as shown in Table S1, Supporting Information.

**Figure 1:**
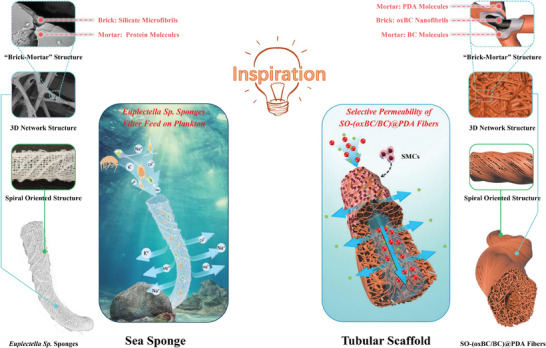
Schematic illustration of *Euplectella*‐inspired bacterial cellulose (BC)‐based fibers as tubular scaffolds.

Nature, through millions of years of evolution, provides us with excellent strategies to learn from, as exemplified by the *Euplectella sp*. sponge, a tubular marine organism.^[^ ^43^, ^44^, ^45^, ^46^ ^]^ This sponge offers a compelling blueprint for tubular scaffold design due to its robust and porous network of microfibers arranged in a spiral orientation, as shown in **Figure** **1**. This structure not only supports efficient material exchange but also withstands strong deep‐sea currents, thanks to its high modulus inorganic silicate fibers and protein‐based “brick‐and‐mortar” composite structure.^[^ ^47^, ^48^, ^49^, ^50^ ^]^ The spiral orientation of these microfibers helps distribute mechanical stress, enhancing the structure's overall durability and making it an excellent model for inducing cell alignment in scaffold designs.

Inspired by the *Euplectella* sponge, we have developed a novel strategy to construct a stable, 3D porous network using oxidized bacterial cellulose (oxBC) nanofibrils combined with bacterial cellulose (BC) and polydopamine (PDA) molecules. This network was further reinforced by an in situ polymerized PDA coating, forming a robust “brick‐and‐mortar” structure. By employing a fluid twisting technique during the microfluidic coaxial spinning process, we achieved a nanoscale spiral orientation of the nanofibrils, effectively mimicking the sponge's natural architecture. This innovative scaffold design not only exhibits high mechanical strength and selective permeability but also supports the spiral alignment of SMCs and the random arrangement of endothelial cells (ECs), making it a promising candidate for constructing realistic tubular organ models. In addition, preliminary tests indicate that these sponge‐like hollow fibers are biocompatible and do not elicit significant immune responses or metabolic issues, highlighting their potential as implantable biomaterials. By leveraging nature‐inspired design principles, our approach addresses the challenges of creating functional, durable, and biocompatible scaffolds for tissue engineering applications.

## 2 Results

**Figure 2:**
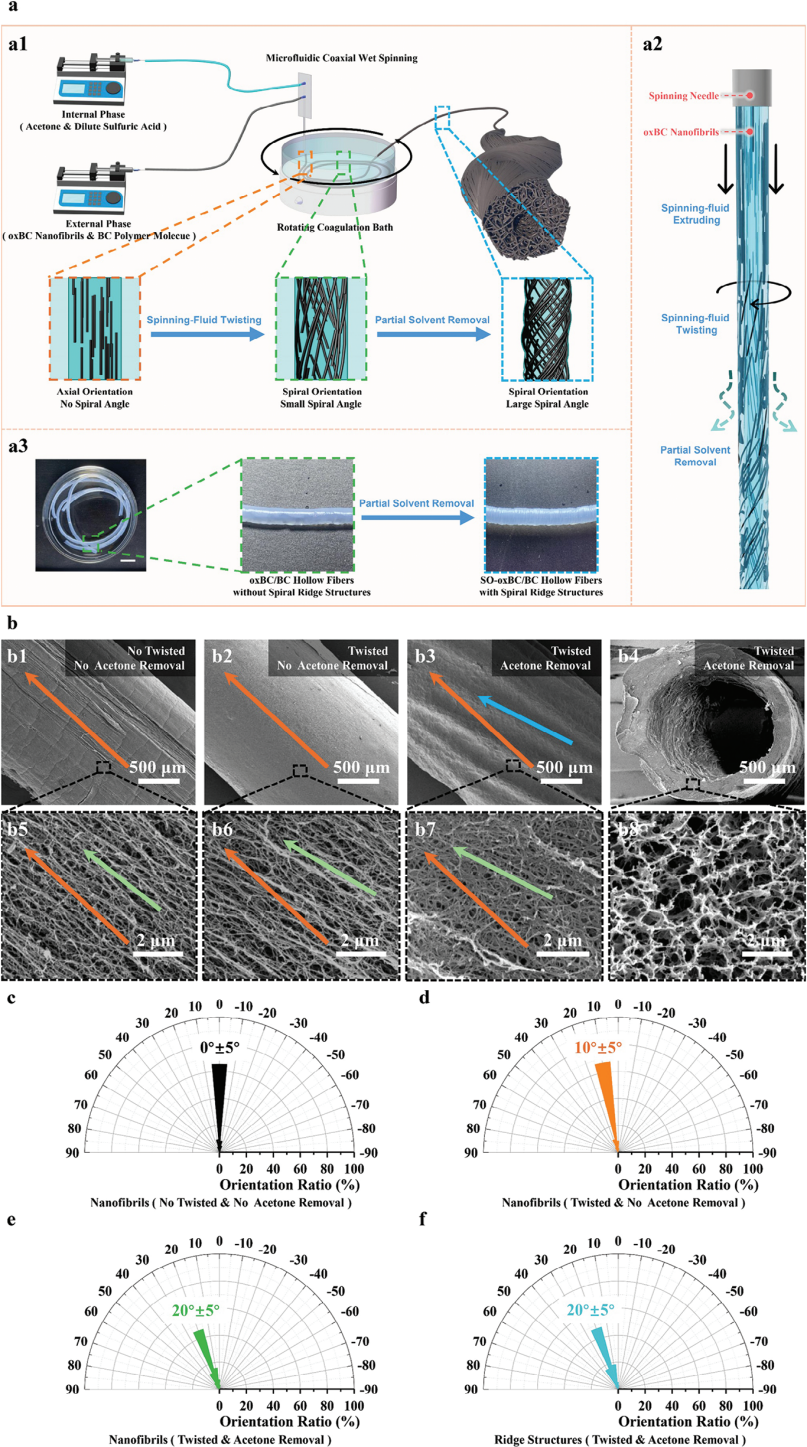
Preparation and morphological characterization of SO‐o*x*BC/BC hollow fibers. a) Preparation and structural evolution of SO‐o*x*BC/BC hollow fibers: a1) Schematic diagram of the preparation process and structural evolution of SO‐o*x*BC/BC hollow fibers; a2) evolution of orientation direction of o*x*BC nanofibrils at various stages of fiber preparation; and a3) optical photograph of the formation of ridge‐like structures on the surface of SO‐o*x*BC/BC hollow fibers. b) SEM images showing the morphology of SO‐o*x*BC/BC hollow fibers after different treatments (orange arrow: the direction of the fiber axis, green Arrow: the mean direction of the oxBC nanofibrils, blue arrow: the mean direction of the ridges): b1) Surface morphology without spinning fluid twisting and without acetone removal; b2) surface morphology with spinning fluid twisting but without acetone removal; b3) surface morphology with both spinning fluid twisting and acetone removal; b4) cross‐section morphology with spinning fluid twisting and acetone removal; and b5–b8) enlarged views corresponding to SEM images (b1–b4). c) Nanofibrils orientation direction of SO‐o*x*BC/BC hollow fibers without spinning fluid twisting and without acetone removal. d) Nanofibrils orientation direction of SO‐o*x*BC/BC hollow fibers with spinning fluid twisting but without acetone removal. e) Nanofibrils orientation direction of SO‐o*x*BC/BC hollow fibers with both spinning fluid twisting and acetone removal. f) Orientation direction of ridge‐like structures of SO‐o*x*BC/BC hollow fibers with spinning fluid twisting and acetone removal.

Figure 1 illustrates the initial synthesis of o*x*BC/BC fibers that mimic the structural composition of *Euplectella sp*. sponges, featuring a porous network and spiral oriented surface microfibers. This was achieved through the integration of microfluidic coaxial wet spinning, spinning fluid twisting, and partial solvent removal techniques (**Figure** **2**b; Figure S1, Supporting Information). In the spinning process (for detailed spinning steps, please refer to the Experimental Section), a spinning dope composed of an aqueous dispersion of o*x*BC nanofibril mixed with BC solution was extruded into a coagulation bath containing acetone and dilute sulfuric acid, as shown in Figure 2a. BC is a natural biopolymer with nanofibril structures tens of nanometers in diameter (see Figure S2, Supporting Information), matching the optimal size for guiding SMCs alignment. In addition, BC nanofibrils have a high modulus and excellent biocompatibility, making them ideal “bricks” for scaffold materials resembling *Euplectella sp*.‐like sponge structures.^[^ ^51^, ^52^ ^]^

While natural BC nanofibrils possess excellent properties, they have poor dispersibility in solvents and limited processability. o*x*BC nanofibrils are water‐dispersible but form structurally unstable fibers in aqueous environments due to their dispersibility, leading to fiber dissociation—a critical issue for tissue engineering scaffolds. To overcome this, we incorporated BC polymer molecules dissolved in water into the o*x*BC nanofibrils dispersion. BC is insoluble in neutral water at room temperature but can be dissolved under low temperature (<−5 °C) and strong alkaline conditions; it remains in solution at room temperature (see Figure S3, Supporting Information). By adjusting the pH to acidic conditions during spinning, the BC molecules precipitate, becoming insoluble and reinforcing the fiber structure.^[^ ^53^ ^]^

During spinning, acetone in the coagulation promotes the formation of a 3D network by oxBC nanofibrils. Simultaneously, dilute sulfuric acid causes the BC polymer molecules to precipitate and solidify, reinforcing the network akin to “mortar” filling gaps between “bricks” (oxBC nanofibrils). The interfacial energy and hydrogen bonding between oxBC nanofibrils and BC molecules facilitate this process. As shown in Figure S4, Supporting Information, by comparing the microscopic morphologies of pure o*x*BC fibers and o*x*BC/BC composite fibers, it can be found that, compared with pure o*x*BC fibers, the network skeleton of oxBC/BC composite fibers is relatively rough. Moreover, there is an obvious residual liquid film structure at the intersection of nanofibers, which is formed by the shrinkage and solidification of the BC solution liquid film in the network skeleton during solvent removal. This indicates that BC molecules will finally adhere to the surface of o*x*BC nanofibrils to form a “brick–mortar” structure. In addition, due to the liquid film retraction effect, BC molecules are more concentrated at the intersection of nanofibers. Therefore, using a mixture of o*x*BC nanofibrils and BC solution in the microfluidic coaxial wet spinning system can produce hollow fibers with a stable 3D network structure (Figure 2b). Further, this “brick–mortar” structure is also beneficial to the stability of the hollow fibers in water. As can be seen from Figure S5, Supporting Information, after shaking the glass bottle, the o*x*BC fibers break in water; while, the o*x*BC/BC composite fibers can maintain their fiber shape. This is because o*x*BC nanofibrils have good water dispersibility, and the network skeleton composed only of o*x*BC nanofibrils is easily dissociated in water; so, pure o*x*BC fibers cannot stably maintain their shape in water. As BC does not dissolve in water under non‐ultra‐low temperature conditions, if BC molecules are coated on the network skeleton composed of o*x*BC nanofibrils, the three‐dimensional network structure in the fibers can be stabilized. The fact that o*x*BC/BC composite fibers can stably exist in water indirectly proves that the o*x*BC/BC composite fibers form a “brick–mortar” structure in which BC molecules coat o*x*BC nanofibrils. This method also allows easy adjustment of the hollow fibers' dimensions, making it efficient and suitable for continuous manufacturing (Figure 2b; Figures S6 and S7 and Movie S1, Supporting Information).

To create a spiral‐oriented structure resembling the surface microfibers of *Euplectella sp*. sponges, we introduce circumferential shear flow to the spinning solution by using a rotating coagulation bath. In conventional spinning systems, nanofibrils align axially due to shear along the fiber axis. However, in our setup, the coagulation bath rotates. When the newly formed fiber extends from the needle to the bottom of the bath, its free end is carried by the rotating bath surface; while, the end near the needle remains relatively stationary, as shown in Figure 2a. This differential movement imparts a torsional (twisting) strain to the fiber, causing the initially axially aligned nanofibers to reorient into a spiral alignment. This process is analogous to yarn twisting. Therefore, we refer to this process as spinning fluid twisting.

The SEM images (Figure 2b5,b6) and statistical analysis (Figure 2c,d) of the orientation direction of nanofibrils within the fibers reveal that, following the twisting of the spinning fluid, the angle (*θ*) between the o*x*BC nanofibrils' orientation and the fiber axis (referred to as the spiral angle) shifts from *θ* ≈ 0° to *θ* > 0°. This confirms the effectiveness of the method in creating spiral‐oriented microstructures. In addition, the SEM images reveal that regardless of whether the spinning fluid is twisted or not, the hollow fiber walls exhibit a similar macroporous network structure formed by nanofibrils. The porosity is as high as 94%, and it has a specific surface area of 163 m^2^ g^−1^, as shown in Figure S8, Supporting Information. This structure supports the permeability of the fiber walls but also results in a loose surface, potentially limiting effective cell adhesion as reducing the contact area between cells and the fiber surface

To address this, the initial hollow fibers were exposed to 50% humidity at room temperature for 25 min. This treatment promoted partial solvent evaporation, leading to densification on the fiber surface; while, retaining the macroporous network structure in the fiber wall, as shown in Figure 2b7,b8. The reduced reduction of pore size and denser accumulation of nanofibrils on fiber surface after solvent removal highlight the effectiveness of this approach. Acetone is the primary solvent being removed because its volatility is much higher than that of water. This allows for easy adjustment of the surface densification degree of the fiber surface by varying the acetone proportion. The removal of solvents increases the spiral angle, with *θ*_1_ > *θ*_0_, where *θ*_0_ and *θ*_1_ represent the nanofibril orientation angles before and after partial solvent evaporation (Figure 2b6,b7,d,e). We hypothesize that this increase results from uneven axial and radial shrinkage induced by the nanofibril orientation. Further, this uneven shrinkage leads to the formation of visible spiral ridges on the fiber surface, whose orientation aligns statistically with the o*x*BC nanofibrils' orientation direction, as depicted in Figure 2b,e,f. This provides great convenience for obtaining the orientation direction of o*x*BC nanofibrils. These fibers, characterized by their spiral orientation, are referred to as SO‐o*x*BC/BC fibers.

**Figure 3:**
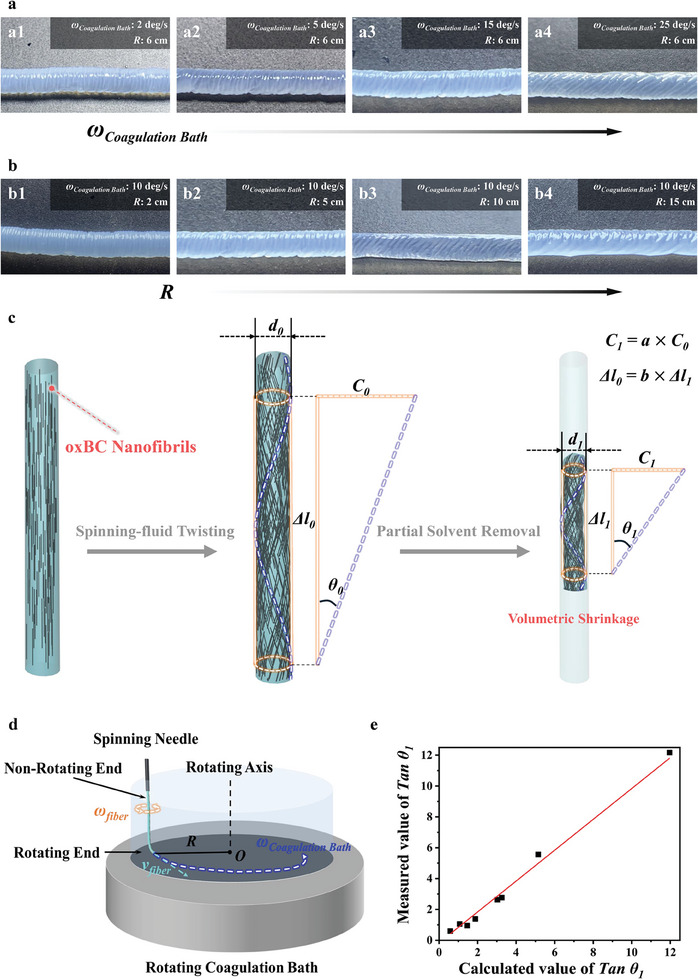
Influence of spinning parameters on the orientation of surface nanofibrils and ridge structures in SO‐o*x*BC/BC hollow fibers ‘c’. a) Optical images of SO‐o*x*BC/BC fibers prepared at different rotational angular velocity (*ω*_coagulation bath_) of the coagulation bath. b) Optical images of SO‐o*x*BC/BC fibers prepared with varying distance (*R*) between the needle and the center of the rotating coagulation bath. c) Schematic illustration of the evolution of nanofibrils orientation direction on the fiber surface before and after spinning fluid twisting and acetone removal (*d*_0_ *, d*_1_ *, c*_0_ *, c*_1_ *, Δl*_0_ *, Δl*_1_ *, θ*_0_, and *θ*_1_ represent the diameter, circumference, pitch, and spiral angle of the fiber before and after volumetric shrinkage, respectively). d) Schematic diagram of the spinning fluid twisting mechanism (*ω*_coagulation bath_ represents the rotational angular velocity of the coagulation bath, *ω*_fiber_ represents the angular velocity of spinning fluid twisting, and *v*_fiber_ represents the speed of fiber growth, that is, the rotational linear velocity of the fiber landing point on the coagulation bath bottom). e) Comparison of theoretical and experimental values of tan *θ*, where *θ* is the angle between the orientation direction of nanofibrils and the fiber axis.

Further investigations demonstrated that the alignment direction of surface ridges, or of o*x*BC nanofibrils on SO‐o*x*BC/BC fibers could be controlled by adjusting the rotational angular velocity of the coagulation bath (*ω*_coagulation bath_) and the distance between the needle and the center of the rotating coagulation bath (*R*), as illustrated in **Figure** **3**a,b. The orientation of o*x*BC nanofibrils resulted from the combined effects of axial shear during the extrusion and circumferential shear from the rotating coagulation bath. As the coagulation bath rotated, it generated a circumferential shear flow. When the spinning fluid entered the bath, its free end rotated under the influence of the circumferential shear flow; while, the end near the needle remained stationary.

This differential rotation caused the fluid to twist, reorienting the o*x*BC nanofibrils from axial to spiral alignment, as illustrated in Figure 3c,d. By deriving and analyzing the relationship between spinning parameters and nanofibril orientation (details in the Supporting Information), we found that the final orientation angle (*θ*_1_) of o*x*BC nanofibrils was positively correlated with the spinning fluid flow rate (*Q*) and inversely correlated with the rotational angular velocity of the coagulation bath (*ω*_coagulation bath_) and the distance between the needle and the center of the rotating coagulation bath (*R*):

(1) $$\tan\theta_{1}=\frac{a}{b}\times \sqrt{\frac{Q}{\pi \times \omega_{\mathrm{coagulation}\;\mathrm{bath}}\times R^{3}}}$$

where *a* and *b* are the shrinkage ratios in the circumferential and axial directions, respectively. Comparing the experimental tan *θ* values of SO‐o*x*BC/BC fibers prepared under different *ω*_coagulation bath_ and *R* parameters with theoretical calculations revealed a close match when the ratio of *a* to *b* was 154.9, yielding a *R^2^* value of 0.995, as shown in Figure 3e. This analytical model accurately predicts the variation trend of the angle under various spinning parameters.

**Figure 4:**
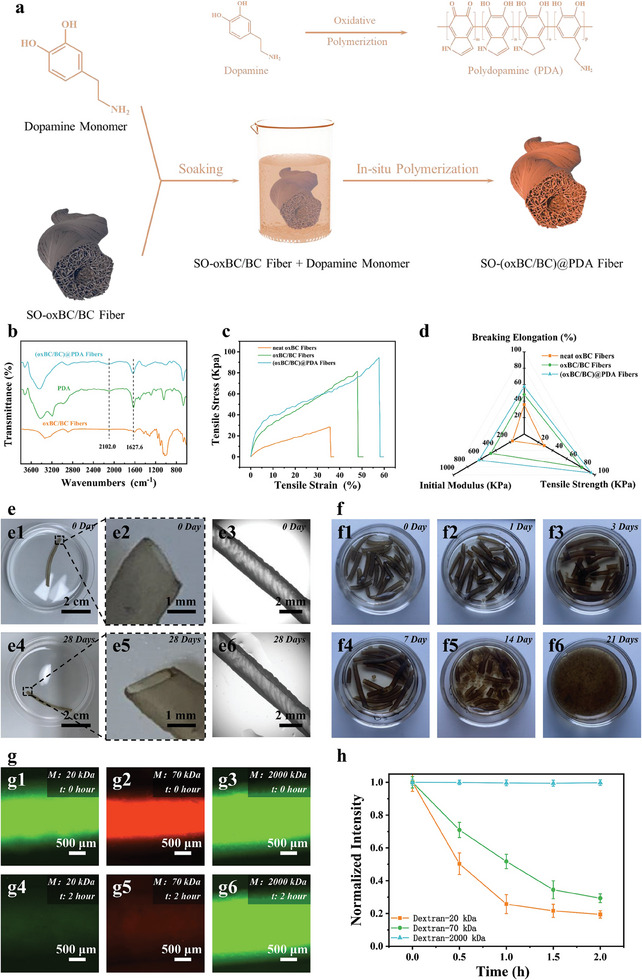
Preparation mechanism and properties of SO‐(o*x*BC/BC)@PDA fibers. a) Schematic illustration of the preparation process for SO‐(o*x*BC/BC)@PDA fibers. b) FTIR spectra of o*x*BC/BC fibers (orange), PDA (green), and (o*x*BC/BC)@PDA fibers (blue). c) Stress–strain curves comparing neat o*x*BC fibers, o*x*BC/BC fibers, and (o*x*BC/BC)@PDA fibers. d) Spider chart illustrating the mechanical properties, including tensile stress, tensile strain, and modulus, of neat o*x*BC fibers, o*x*BC/BC fibers, and (o*x*BC/BC)@PDA fibers. e) Optical and microscopic images of SO‐(o*x*BC/BC)@PDA fibers soaked in water for 0 and 28 days: e1–e3) images at 0 days and e4–e6) images at 28 days. f) Images of SO‐(o*x*BC/BC)@PDA fibers after enzymatic hydrolysis for various durations (0, 1, 3, 7, 14, and 21 days). g) Fluorescence images showing the diffusion of FITC‐dextrans with different molecular weights (20, 70, and 2000 kDa) inside the SO‐(o*x*BC/BC)@PDA fibers after 0 and 2 h. h) Normalized fluorescence intensity of FITC‐dextrans over time, indicating the permeability of the fibers to molecules of different sizes.

To enhance cell adhesion and network structure further, a second “mortar,” polydopamine (PDA), was loaded onto the 3D network skeleton surface of fibers using in situ polymerization, resulting in SO‐(o*x*BC/BC)@PDA fibers, as depicted in **Figure** **4**a. PDA, a widely used bioadhesive, transformed fiber color from white to dark gray upon successful loading (Figure S9, Supporting Information). Characteristic peaks at 1627.6 and 2102.0 cm^−1^ in the FTIR spectrum confirmed PDA loading, indicating N─H bending and C═C stretching vibrations in the PDA aromatic ring (Figure 4b).

Hollow fibers with thin walls and highly porous 3D networks in the tube walls often exhibit extremely poor mechanical properties. The mechanical test results of fibers composed solely of o*x*BC nanofibrils (neat oxBC fibers) also confirm this, as shown in Figure 4c,d. However, the “brick/mortar/mortar” composite structure composed of o*x*BC nanofibrils/BC polymer molecules/PDA polymer molecules endows SO‐(o*x*BC/BC)@PDA hollow fibers with improved mechanical properties. Compared with neat o*x*BC fibers, the tensile strength (188.2%), elongation at break (34.5%), and initial modulus (185.6%) of SO‐o*x*BC/BC hollow fibers are all increased, as shown in Figure 4c,d. Further reinforcement with PDA increases these properties by 234.1%, 61.6%, and 287.4%, respectively, compared with neat o*x*BC fibers. The mechanical strength of SO‐(o*x*BC/BC)@PDA hollow fibers increases to 94.3 kPa, which is within or above the range of many soft scaffolds made from common biopolymers such as collagen, gelatin, or hyaluronic acid. The systolic blood pressure of a healthy individual is usually lower than 18.7 kPa (140 mmHg), and even that of a hypertensive patient usually does not exceed 39.9 kPa (300 mmHg). Therefore, under hypertensive conditions, when SO‐(o*x*BC/BC)@PDA hollow fibers are used as vascular scaffolds, their strength exceeds the upper limit of blood pressure load by at least twice, and under normal blood pressure conditions, it exceeds about five times. SO‐(o*x*BC/BC)@PDA hollow fibers also possess the characteristics of being knotable, excellent swelling resistance, and self‐supporting ability (Figures S10 and S11, Supporting Information; Figure 4e). Even after being immersed in water for 28 days, these hollow fibers with a wall thickness of only ≈180 µm maintain their structure without significant deformation or collapse, in sharp contrast to hydrogel tubular fibers that are prone to swelling. Remarkably, while having excellent structural stability, the modulus of SO‐(o*x*BC/BC)@PDA fibers is not too high and overlaps well with the reported values of natural blood vessels ranging from 10^2^ to 10^3^ kPa, which eliminates concerns about scaffold compliance. Being entirely composed of bio‐based materials, SO‐(o*x*BC/BC)@PDA hollow fibers are readily degradable by enzymes. After 3 weeks of enzymatic treatment with cellulase, the macroscopic structure of the hollow fibers was completely disintegrated as depicted in Figure 4f. By further analyzing the mass loss at different times, the results show that under this enzymatic hydrolysis condition, the SO‐(o*x*BC/BC)@PDA hollow fibers exhibited a degradation behavior conforming to first‐order kinetics, as shown in Figure S12, Supporting Information. Using the first‐order kinetics equation (see Equation (S2), Supporting Information) and linear fitting, the degradation rate constant of the SO‐(o*x*BC/BC)@PDA hollow fibers was determined to be 0.033. In addition, previous research has indicated that the degradation rate of cellulose is closely related to enzyme concentration, allowing for controlled degradation of SO‐(o*x*BC/BC)@PDA hollow fibers. Controlled degradation is particularly crucial for tissue engineering scaffolds, where the scaffold needs to be removed as the cultured tissue matures.

SO‐(o*x*BC/BC)@PDA hollow fibers, resembling tubular organ structures, featured a porous tube wall and hollow interior. Injecting dye solutions into one end demonstrated smooth flow out the opposite end, highlighting the fibers' fluid transport capability and potential for simulating tubular organ functions (Figure S13 and Movie S2, Supporting Information). Moreover, injecting fluorescently labeled dextran solutions of varying molecular weights into fiber cavities and studying diffusion behavior through fiber walls revealed selective permeability. Dextran molecules of 70 kDa diffused quickly through abundant pores in SO‐(o*x*BC/BC)@PDA fiber walls, whereas higher molecular weight dextran (2000 kDa) remained confined within fiber cavities, as depicted in Figure 4g,h. This selective permeability mirrored the substance exchange requirements of tubular organs, ensuring substances such as glucose, oxygen, carbon dioxide, and inorganic salts (typically below 70 kDa) could pass through freely. It is worth mentioning that this spinning method could easily adjust the parameters such as the flow rate, the speed of the coagulation bath, and the solvent removal process to change the pore structure of the SO‐(o*x*BC/BC)@PDA hollow fibers, thereby meeting the different permeability requirements of various tissue engineering applications.

**Figure 5:**
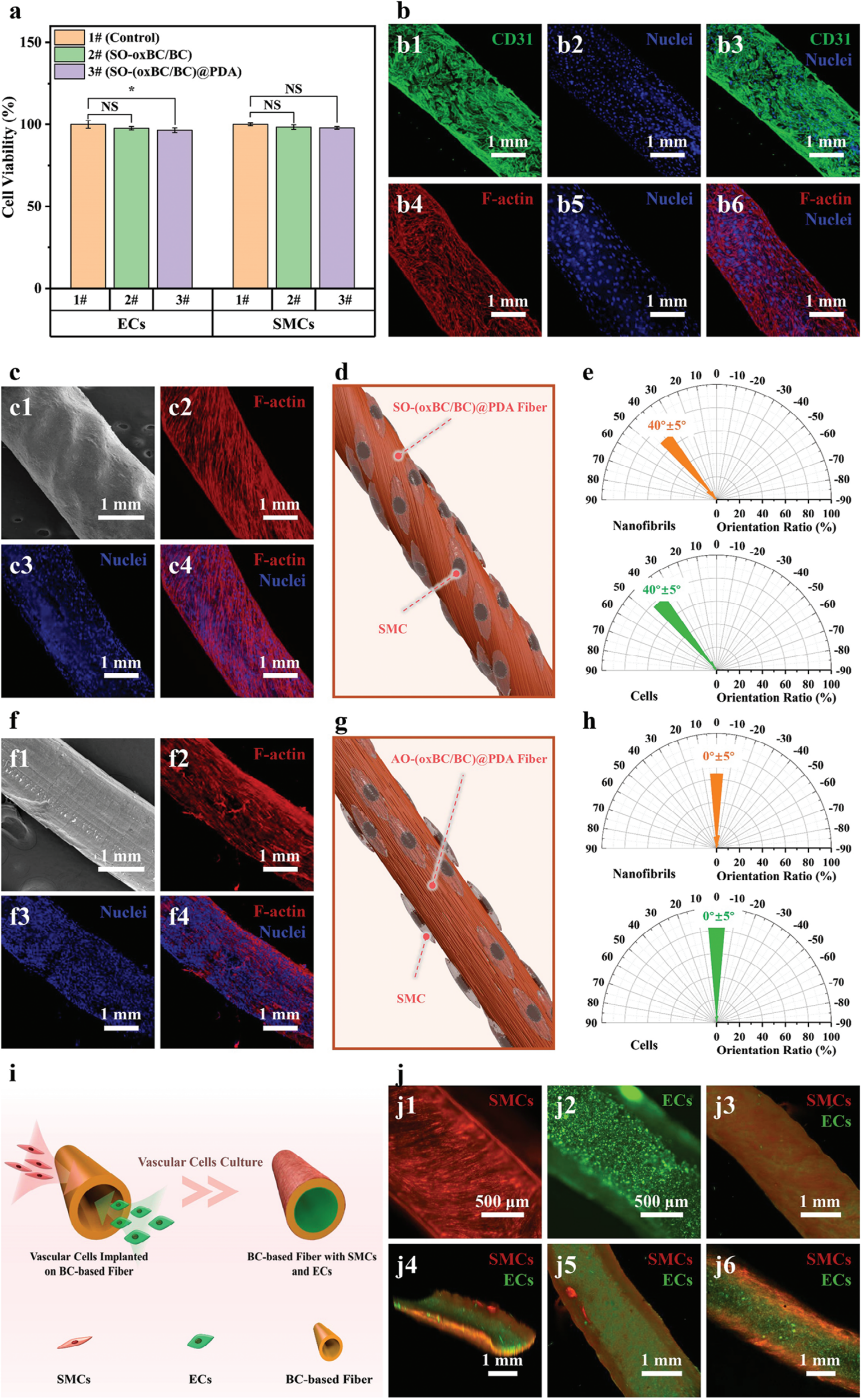
Cultivation of vascular cells on SO‐(o*x*BC/BC)@PDA hollow fibers. a) Cell viability of ECs and SMCs co‐cultured with SO‐o*x*BC/BC and SO‐(o*x*BC/BC)@PDA fibers. b) Fluorescence images of ECs on SO‐(o*x*BC/BC)@PDA fibers: b1–b3) CD31 and b4–b6) F‐actin. c) Images of SMCs on SO‐(o*x*BC/BC)@PDA fibers: c1) SEM images showing spiral ridge structures and c2–c4) fluorescence images of F‐actin expression in SMCs. d) Schematic diagram of SMCs orientation on SO‐(o*x*BC/BC)@PDA fibers. e) Comparison of the orientation direction of o*x*BC nanofibrils and SMCs on the fiber surface. f) Images of SMCs on AO‐(o*x*BC/BC)@PDA fibers: f1) SEM images without spiral ridge structures and f2–f4) fluorescence images of F‐actin expression in SMCs. g) Schematic diagram of SMCs orientation on AO‐(o*x*BC/BC)@PDA fibers. h) Comparison of the orientation direction of o*x*BC nanofibrils and SMCs on AO‐(o*x*BC/BC)@PDA fibers. i) Schematic diagram of co‐culturing ECs and SMCs on the inner and outer surfaces of SO‐(o*x*BC/BC)@PDA fibers. j) Fluorescence images of co‐cultured cells: j1) SMCs on the outer surface; j2) ECs on the inner surface; j3) confocal images of SMCs; j4) 3D reconstruction of co‐cultured cells; j5) confocal images of ECs; and j6) cross‐sectional view of the 3D reconstruction.

To validate the potential of SO‐(o*x*BC/BC)@PDA hollow fibers as scaffold materials for in vitro models of tubular organs, we conducted comprehensive performance studies using vascular models as an example. Initially, SO‐o*x*BC/BC fibers and SO‐(o*x*BC/BC)@PDA fibers were co‐cultured with ECs and SMCs. As certain reagents (such as dilute sulfuric acid and acetone) used in the fiber manufacturing process can be harmful to cells, the fibers were thoroughly rinsed with deionized water multiple times in advance before the co‐culture experiment to remove any residual solvents. After these rinses, we replaced the water in the fibers with phosphate‐buffered saline (PBS) before introducing the cells. The specific steps are described in the Experimental Section. Both types of fibers exhibited excellent biocompatibility. Although the *p*‐value of the experimental group co‐cultured with SO‐(o*x*BC/BC)@PDA fibers was between 0.01 and 0.05 compared with the control group, which indicates that SO‐(o*x*BC/BC)@PDA fibers may affect the activity of endothelial cells to some extent, the survival rate of endothelial cells in this experimental group also exceeded 95%. Therefore, SO‐(o*x*BC/BC)@PDA fibers were still considered as scaffolds for endothelial cell growth, as shown in **Figure** **5**a. However, significant differences were observed in cell adhesion properties between the two fiber types. Under identical co‐culture conditions, minimal adhesion of ECs or SMCs was observed on SO‐o*x*BC/BC fibers, whereas SO‐(o*x*BC/BC)@PDA fibers exhibited dense coverage by ECs or SMCs, as shown in Figure S14, Supporting Information. This disparity is attributed to the abundant negatively charged functional groups on the o*x*BC surface, resulting in a high negative potential that repels similarly charged cells via electrostatic repulsion. In contrast, PDA, with its positively charged functional groups, neutralizes the negative potential on fiber surfaces, promoting cell adhesion and growth on SO‐(o*x*BC/BC)@PDA fibers. Hence, SO‐(o*x*BC/BC)@PDA hollow fibers are considered more suitable as biological scaffold materials.

Further investigations revealed that after 3 days of EC culture on SO‐(o*x*BC/BC)@PDA hollow fibers, confocal microscopy images showed co‐expression of filamentous actin (F‐actin) and CD31 (an endothelial cell‐cell adhesion marker), indicating tight cell–cell contacts and the formation of a complete endothelial monolayer‐endothelialization (Figure 5b). Endothelialization is crucial for imparting antithrombotic, barrier, and selective permeability functions in vascular models. The expression of F‐actin in SMCs indicated the formation of a dense smooth muscle layer on the surface of SO‐(o*x*BC/BC)@PDA fibers, as displayed in Figure 5c. The filamentous F‐actin in SMCs showed a significant spiral alignment along the SO‐(o*x*BC/BC)@PDA fibers, aligning with the elongation direction of SMCs. This indicates that the SO‐(o*x*BC/BC)@PDA hollow fibers successfully induced the SMCs to align in a spiral direction (Figure 5c,d). Detailed studies showed that SMCs aligned at an angle of 40° ± 5° relative to the fiber axis, with an alignment rate of 66.7% (Figure 5e). The alignment direction of SMCs matched the orientation of the spiral ridge structures or o*x*BC nanofibrils on the fiber surface.

For comparative analysis, we prepared AO‐(o*x*BC/BC)@PDA fibers with nanofibrils oriented along the fiber axis using a static coagulation bath. SMCs cultured on AO‐(o*x*BC/BC)@PDA fiber surfaces also exhibited significant alignment but strictly along the fiber axis, consistent with the orientation of o*x*BC nanofibrils in these fibers (Figure 5f,h). These findings highlight the strong inductive effect of nanofibril alignment on SO‐(o*x*BC/BC)@PDA fiber surfaces in directing SMC alignment. This suggests that the alignment direction of SMCs can be precisely regulated by altering the orientation direction of the o*x*BC nanofibrils on the surface.

Subsequently, ECs and SMCs were separately seeded on the inner and outer surfaces of SO‐(o*x*BC/BC)@PDA hollow fibers and co‐cultured for a week (Figure 5i). Confocal microscopy confirmed that co‐cultured SMCs (red fluorescence) and ECs (green fluorescence) formed confluent layers on the outer and inner surfaces of the fibers, respectively (Figure 5j). SMCs on the outer fiber surface exhibited regular spiral alignment, whereas ECs on the inner fiber surface formed tightly connected, non‐oriented arrangements. This spatial distribution and cellular arrangement closely mimic natural blood vessels. Successful co‐culture of these vascular cell types with normal phenotypes, combined with properties such as anti‐swelling, enzyme degradation, injectability, and wall permeability, underscores the potential application of SO‐(o*x*BC/BC)@PDA hollow fibers as scaffold materials in in vitro models of tubular organs such as blood vessels.

**Figure 6:**
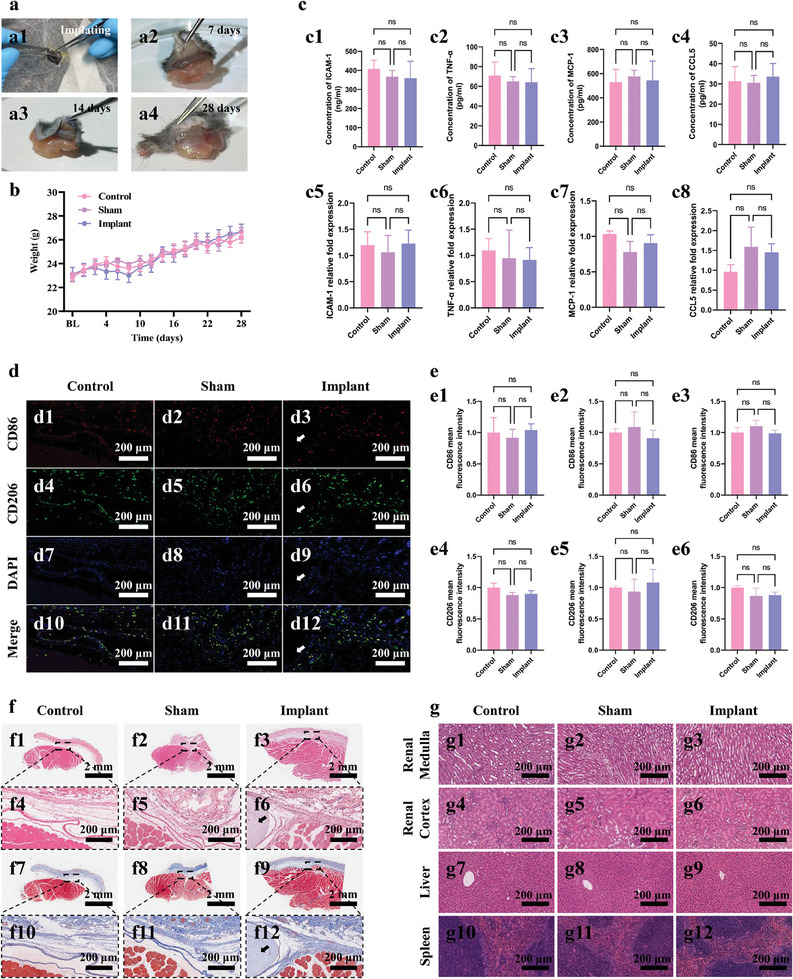
Physiological evaluation of mice after implantation of SO‐(o*x*BC/BC)@PDA fibers. a) Images of the implantation site at various time points post‐implantation: a1) implanting; a2) 7 days; a3) 14 days; and a4) 28 days. b) Body weight changes of mice over time after implantation. c) Comparison of inflammatory cytokine levels (ICAM‐1, TNF‐α, MCP‐1, and CCL5) at implantation site among control group, sham group, and implant group, measured by c1–c4) ELISA and c5–c8) PCR. d) Fluorescence images of macrophages at the implantation site 28 days post‐implantation, stained for CD86 (red), CD206 (green), and nucleus (blue). Arrows indicate the implanted fibers. e) Comparison of CD86 and CD206 expression in tissue sections from control, sham, and implant groups at e1,e4) 7 days, e2,e5) 14 days, and e3,e6) 28 days post‐implantation. f) Microscopic images and enlarged view of tissue sections stained with: f1–f6) Hematoxylin and eosin (HE) or f7–f12) Masson's trichrome, showing the implantation site at 28 days. Arrows indicate the implanted fibers. g) Microscopic images of HE‐stained tissue sections of g1–g3) renal medulla, g4–g6) renal cortex, g7–g9) liver, and g10–g12) spleen, 28 days post‐implantation.

We also verified the potential of SO‐(o*x*BC/BC)@PDA fibers as implantable biomaterials. Surgical trauma, post‐implantation tissue stimulation, and scaffold toxicity can trigger immune responses. We employed a minimally invasive subcutaneous implantation method to implant SO‐(o*x*BC/BC)@PDA hollow fibers into mice, minimizing surgical trauma interference and highlighting the impact of the scaffold material itself. Scaffolds with poor biocompatibility or immunotoxicity can cause severe inflammation, tissue damage, metabolic abnormalities, weight loss, and potentially death in implanted mice. Mice in the control group (untreated), sham‐operated group (surgery without scaffold implantation), and implantation group (scaffold‐implanted) were all fed under the same conditions for 28 days and remained healthy with no significant differences in body weight, as shown in **Figure** **6**a,b. Photographs of the tissue surrounding the implantation site showed no obvious redness, swelling, or hyperplasia.

Seven days post‐surgery, we homogenized the tissue at the wound site and analyzed the expression of inflammatory factors using ELISA and PCR (Figure 6c). The expression levels of TNF‐α, ICAM‐1, MCP‐1, and CCL5in the tissue homogenate supernatant showed no statistically significant differences among the groups, suggesting that surgical trauma and scaffold implantation did not lead to significant recruitment of inflammatory factors by day 7.

Macrophage polarization, particularly M1 and M2 phenotypes, plays a crucial role in an effective immune response. To evaluate macrophage polarization after implantation, tissue sections from control, sham‐operated, and implantation groups were stained for the M1 macrophage marker CD86 and M2 macrophage marker CD206, as shown in Figure 6d; Figure S15, Supporting Information. Quantification of fluorescence indicated that the expression levels of CD86 and CD206 in the sham‐operated and implantation groups were statistically indistinguishable from those in the control group (Figure 6e), indicating that surgical trauma and scaffold fiber implantation did not induce significant macrophage polarization or severe inflammatory response.

To further characterize the immune response at different stages post‐implantation, tissue sections from the implantation site were analyzed at days 7, 14, and 28 post‐implantation (Figure 6f; Figures S16 and S17, Supporting Information). HE and Masson's trichrome staining showed no significant differences in immune cell infiltration among the groups, indicating no obvious inflammation at any observed time point. Further, BC and its derivatives gradually degraded in vivo. Shrinkage and deformation of the scaffold fibers observed in tissue sections from the implantation group at days 14 and 28 confirmed this biodegradability, meeting the requirements for tissue engineering scaffolds. To ensure the biosafety of the degradation products, we examined tissue sections of the metabolic organs (kidneys and liver) and immune organs (spleen) of experimental mice at 28 days post‐implantation. Figure 6g shows no abnormalities in the kidney medulla, kidney cortex, liver, and spleen of mice in the implantation group, sham‐operated group, and control group, indicating that the degradation products of SO‐(o*x*BC/BC)@PDA hollow fibers were non‐immunotoxic and easily metabolized. In conclusion, SO‐(o*x*BC/BC)@PDA hollow fibers and their degradation products exhibited excellent biocompatibility and biosafety, making them suitable for applications as implantable biomaterials. While our study focused primarily on vascular tissue engineering, the underlying principles—coaxial spinning, rotating coagulation bath, and controlled nanofibril orientation—could be adapted for a variety of tubular organs such as the trachea and intestines. For tracheal tissue engineering, tubular scaffolds that mimic the native structure can greatly facilitate epithelial and cartilage regeneration. Adjusting scaffold diameter, rigidity, and surface chemistry may yield constructs suitable for airway repair.^[^ ^54^ ^]^ Similarly, intestinal applications demand tubular scaffolds with specific mechanical and topographical cues to support enteric cell growth and functionality; our fabrication process could be fine‐tuned to meet these requirements.^[^ ^55^ ^]^ By modifying parameters such as scaffold diameter, wall thickness, and surface functionalization (e.g., with growth factors or adhesive peptides), investigators could tailor the mechanical properties and biological cues for various organ systems. In addition, the “brick‐and‐mortar” arrangement of aligned nanofibrils has been shown to guide cell organization, an advantage that might be harnessed for tissue‐specific patterns of growth and regeneration in multiple organ types.

## 3 Conclusion

We have developed an innovative approach to fabricate biomimetic hollow fibers with nanoscale spiral microstructures on their surface, effectively inducing the spiral orientation of SMCs. By precisely controlling the shear rate during the microfluidic coaxial spinning process and utilizing bacterial cellulose to simulate the collagen nanofibrils, we created a scaffold that closely mimics the natural extracellular matrix. The introduction of a PDA coating forms a “brick‐and‐mortar” structure, enhancing the stability and applicability of the hollow fiber scaffold and significantly improving cell adhesion. By tailoring the shape of the hollow fiber scaffold, co‐culturing ECs and SMCs on the inner and outer surfaces, and controlling the orientation of nanofibrils on the scaffold's surface, we achieved precise control over the macroscopic structure, cell assembly, and cell orientation in vascular models. In addition, the resulting hollow fibers met the essential requirements for tubular organ scaffold materials, including stability, degradability, mass transfer capability, biocompatibility, and low immunogenicity. Although we did not conduct a comprehensive and long‐term in vivo study in this work, the existing evidence strongly suggests that the SO‐(o*x*BC/BC)@PDA scaffold holds great promise for wide application in tissue engineering. They represent a promising tool in the field of regenerative medicine, offering new avenues for tissue engineering applications and the development of functional artificial organs.

## 4 Experimental Section

### Chemicals and Reagents

Bacterial cellulose (BC) was purchased from Hainan Yide Food Co., Ltd., China. Sodium hydroxide (NaOH), urea (CO(NH_2_)_2_), thiourea (CH_4_N_2_S), 2,2,6,6‐Tetramethylpiperidine 1‐oxyl (TEMPO), sodium bromide (NaBr), sodium hypochlorite (NaClO), sodium chloride (NaCl), Dopamine (DA), tris buffer, sulfuric acid (H_2_SO_4_), acetone (CH_3_COCH_3_), dopamine (C_8_H_11_NO_2_), hydrochloric acid (HCl), *tert*‐butanol (C_4_H_10_O), glycerol, and bovine serum albumin (BSA) were purchased from Aladdin Biochemical Technology Co., Ltd., China. Glucan (20, 70, and 2000 kDa) with fluorescent group was purchased from Sigma–Aldrich Co., Ltd., Germany. Phosphate buffer saline (PBS), trypsin, cell‐tracker green CMFDA, and cell‐tracker red CMTPX were purchased from Thermo Fisher Scientific Co., Ltd., America. Primary human umbilical vein endothelial cells (HUVES), primary human aortic smooth muscle cells (HASMC), and their supernatant were purchased from Guangzhou Xinyuan Co., Ltd., China. Cell counting kit‐8 (CCK‐8) was purchased from Beyotime Biotechnology Co., Ltd., China. Calcein AM/PI cell double staining kit, TritonX‐100, and Tween were purchased from Beijing Solarbio Science & Technology Co., Ltd., China. CD31 mouse monoclonal antibody, smooth muscle actin rabbit polyclonal, CyTM2‐conjugated AffiniPure Donkey Anti‐Mouse lgG(H+L), and CoraLite594‐conjugated Goat Anti‐Rabbit lgG(H+L) were purchased from Proteintech Co., Ltd., America. 4′, 6‐diamidino‐2‐phenylindole (DAPI) was purchased from Biosharp Co., Ltd., China. Neutral cellulase and β‐ glucosidase were purchased from Shenzhen Dehua Biotechnology Co., Ltd. and Yuanye Biotechnology Co., Ltd. respectively.

### Animals

All animal care and protocols were approved by the Laboratory Animal Ethics Committee of Shenzhen University and Guangdong Medical Laboratory Animal Center (NO. C202211‐10). For in vivo experiments, wild‐type C57BL/6J male mice (22–24 g, 8 weeks) were purchased from the Guangdong Medical Science Experiment Center. The animals were housed in the animal facility of Shenzhen University and maintained with a 12‐h light/dark cycle and libitum access to chow and water. The animal holding areas were under constant monitoring. Animals were experimented in strict accordance with the standards in the Guide for the Care and Use of Laboratory Animals of the National Institutes of Health.

### Pretreatment of BC

First, the purchased bacterial cellulose particles were introduced into 1% NaOH solution and boiled for 1 h to remove the residues from bacterial fermentation. These BC particles were then rinsed with deionized water several times until neutral. Next, the washed BC particles were shredded into fine particles using a masher; then, transferred to a homogenizer for further grinding into finer flocs—these BC flocs were BC nanofilaments. Most of the water in the BC floc dispersion was then filtered out by squeezing with a filter cloth, yielding wet BC nanofibrils containing a small amount of water. The moisture content of these wet BC nanofilaments could be determined by drying and weighing. These wet BC nanofilaments could be stored for long‐term in a refrigerator at 4 °C.

### Preparation of BC Solution by Low‐Temperature Alkali‐Dissolution Method

First, the pretreated wet BC nanofibrils were uniformly dispersed in a mixed solution of sodium hydroxide (NaOH), urea (CO(NH_2_)_2_), thiourea (CH_4_N_2_S), and deionized water using tip ultrasonic treatment and stirring for 30 min. The mass ratio was m_bc_: m_NaOH_: m_CO(NH2)2_: m_CH4N2S_: m_H2O_ = 1:7:7:6:79, resulting in a 1 wt% BC concentration. The obtained BC dispersion was then placed in a refrigerator at −20 °C to cool. After 30 min, the low temperature BC dispersion was taken out and stirred in an ice water bath for 20 min. These cooling and stirring steps were repeated several times until the cloudy BC dispersion turned into a transparent BC solution.

### Preparation of o*x*BC Dispersion by a TEMPO‐Oxidation Method

First, the pretreated wet BC nanofibrils (solid content: 1g) were uniformly dispersed in a mixed solution of TEMPO (0.016 g), NaBr (0.1 g), and deionized water (100 mL) using tip ultrasonication and stirring for 30 min. Then, NaClO aqueous solution with a concentration of 3 mmol g^−1^ was added three times at room temperature to initiate the oxidation of BC nanofilaments. The total volume of NaClO aqueous solution added was 4.2 mL. During the oxidation reaction, 1 mol L^−1^ sodium hydroxide aqueous solution was slowly added into the reaction system to keep the pH value of the system between 10 and 11. After 1.5 h of oxidation, the BC nanofilaments were oxidized into o*x*BC nanofilaments. These oxBC nanofilaments were collected by vacuum filtration and washed to neutral pH using deionized water. Finally, dry and clean oxBC nanofilaments were obtained through freeze‐drying.

### Spinning of the o*x*BC/BC Hollow Fibers

The o*x*BC/BC hollow fibers were prepared by a microfluidic coaxial wet spinning method. In this process, a homogeneous mixture of o*x*BC nanofilaments and BC aqueous solution with the equal mass ratios was prepared as described earlier. This mixture served as the outer phase fluid for coaxial spinning. The inner phase fluid consisted of 0.25 mol L^−1^ dilute sulfuric acid and acetone in a 1:1 volume ratio. Both fluids were injected into a coagulation bath through a coaxial needle by two syringe pumps. The internal phase flow rate was 800 µL min^−1^; while, the external phase flow rate was 700 µL min^−1^. The coaxial needle had an inner diameter of 0.4 mm for the small needle and 2.4 mm for the large needle. The coagulation bath contained the same mixture as the inner phase fluid. Through this process, the outer phase mixture of o*x*BC and BC transformed into hollow fibers. These fibers were collected and placed in an environment with 50% humidity at room temperature for 30 min. Finally, the hollow fibers were stored in 0.25 mol L^−1^ dilute sulfuric acid.

### In Situ Loading of PDA on o*x*BC/BC Hollow Fibers

First, 0.5 g o*x*BC/BC hollow fibers were removed from dilute sulfuric acid and rinsed with deionized water until neutral. These fibers were then immersed in 100 mL of a mixture solution containing dopamine (1 mg mL^−1^) and tris buffer (10 mmol L^−1^). The pH of the reaction system was adjusted to 7 using 1 mol L^−1^ hydrochloric acid. The reaction was carried out at room temperature for 12 h, with gentle shaking and protection from light. This process allowed polydopamine to synthesize and load onto the fiber skeleton. Finally, the fibers were thoroughly rinsed with deionized water to remove any unbound dopamine and polydopamine.

### Samples Characterization

A Thermo Scientific Nicolet 6700 type Fourier transform infrared spectrometer (FTIR, America) was used to characterize the surface functional groups of BC nanofibrils, o*x*BC nanofibrils, o*x*BC/BC fibers, and (o*x*BC/BC)@PDA fibers. The carboxyl group content on the surface of the o*x*BC nanofibrils was measured using alkali solution titration and a METTLER SG23 solution conductivity measurement (America). SEM images of all samples were acquired using a Thermo Scientific APREO S field emission scanning electron microscope (America). Prior to imaging, the SEM samples were freeze‐dried by a SCIENTZ 18N/A Vacuum Freezing Dryer (China) and gold‐coated by a Leica ACE600 High Vacuum Magnetron Sputtering Coater (Germany). The mechanical properties of all fiber samples were measured using a Xinfang YG004 fiber strength tester (China). Optical and fluorescent microscope images of all samples were captured using a Nikon Ti2‐E fluorescence microscope (Japan) and a Nikon AX confocal microscope (Japan).

### Statistical Analysis

The combination of ImageJ and Photoshop was used to statistically analyze the orientation direction of o*x*BC nanofibrils, ridge‐like structures, and F‐actin of SMCs. For data with Gaussian distributions, differences between means of multiple groups were analyzed by one‐way ANOVA or two‐way repeated ANOVA, followed by the Tukey or Bonferroni post hoc test. For data without Gaussian distributions, Kruskal Wallis test was employed. A *p‐*value less than or equal to 0.05 was deemed statistically significant. The fit degree and bar charts were generated by Origin.

For detailed information on the reagents, materials, and testing methods employed, please refer to the Supporting Information.

## Conflict of Interest

The authors declare no conflict of interest.

## Supporting information



Supporting Information

Supplemental Movie 1

Supplemental Movie 2

## Data Availability

The data that support the findings of this study are available on request from the corresponding author. The data are not publicly available due to privacy or ethical restrictions.
