# Supplementary material for: Construction of a Biomimetic Tubular Scaffold Inspired by Sea Sponge Structure: Sponge‐Like Framework and Cell Guidance
Source: Adv Sci (Weinh). 2025 Feb 25;12(16):2416627. doi: 10.1002/advs.202416627 (PMC12021052; doi:10.1002/advs.202416627)
Supplement: Supplementary file 1 — Supporting Information [file ADVS-12-2416627-s001.docx]

Supplementary Materials for

**Construction of a Biomimetic Tubular Scaffold Inspired by Sea Sponge Structure: Sponge-Like Framework and Cell Guidance**

*Si Meng^1^, Nihuan Wu^2^, Jie Fang^2^, Yidan Yu^1^, Xin Tang^1^, Yihan Wang^2^, Xiaokang Deng^1^, Cheng Qi^3^, Tiantian Kong^2 ,4^, Tengda Ding^1,*^, Zhou Liu^1,*^*

*Corresponding author. E-mail: dingtengda@szu.edu.cn; zhouliu@szu.edu.cn

**This PDF file includes:**

Supplementary Text

Figures S1 to S17

Table S1

**Other Supplementary Materials for this manuscript include the following:**

Movies S1 to S2

Supplementary Text

**Materials and Methods**

**Determination of carboxyl content:** The carboxyl content of oxBC was measured by conductivity titration to characterize the oxidation degree of BC. Typically, 0.3 g freeze-dried oxBC nanofilaments were uniformly dispersed into 55 ml deionized water by the method of tip ultrasonic and stirring for 30 min in the ice water bath. Then, 5 ml of 0.01 mol/L NaCl aqueous solution was added to oxBC nanofilaments dispersion and stirred evenly. By adding 0.1 mol/L HCl aqueous solution, the pH value of oxBC nanofilaments dispersion was adjusted to 2.5-3.0. Subsequently, 0.04 mol/L NaOH solution was dripped into oxBC nanofilaments dispersion at the rate of 0.15 ml/min by alkali titration. The conductivity of oxBC nanofilament dispersion was measured and recorded every 1min. These data are plotted as a scatter plot of the relationship between time and conductivity of oxBC nanofilament dispersion. Finally, the carboxyl content on oxBC nanofilament can be calculated according to the scatter diagram and the following formula.

*W*_carboxyl content_ = *c* × *(V*_2_*-V*_1_*) /m* (S1)

Where *W*_carboxyl content_ is the carboxyl content on oxBC nanofilament; *c* is the concentration of NaOH solution (in this study, c=0.04 mol/L); *V*_1_ is the volume of NaOH solution consumed at the beginning point of the platform period in the scatter diagram;*V*_2_ is the volume of NaOH solution consumed at the end point of the platform period in the scatter diagram; *m* is the mass of freeze-dried oxBC nanofilaments (in this study, m=0.3 g) *.*

**Cell culture and passage:** HUVES and HASMC were cultured in their respective specialized culture media supplemented with appropriate additives. When cells reached 90% confluency, cell passage was performed. Firstly, the depleted culture media was discarded, and cells were gently washed twice with PBS. Then, 0.125% trypsin was added at room temperature for 1 minute, followed by removal of trypsin and incubation of cells in a CO_2_ cell culture incubator for 2 minutes. After taking out the cells, the digestion was terminated by adding culture medium, and cells were dispersed evenly by pipetting before passage. The cells were then suspended in fresh culture medium for reseeding.

**Cytotoxicity experiments:** oxBC/BC fibers and (oxBC/BC)@PDA fibers were washed with deionized water until neutral, and then immersed in PBS buffer to remove moisture from the fibers. Subsequently, the fibers soaked in PBS were subjected to high-pressure sterilization at 121°C for 20 minutes. Under conditions of 37°C and 5% CO_2_ humidified air, HUVES and HASMC were separately cultured in primary endothelial and smooth muscle culture media. Then, HUVES and HASMC were seeded in a 96-well plate at a density of 5×10^3^ cells per well. After 24 hours of adherent growth in 100 μl of culture medium, 2 cm long BC/OxBC fibers and (oxBC/BC)@PDA fibers were added to the 96-well plate, followed by co-culture for 48 hours. Subsequently, fresh culture medium was replaced and cck-8 reagent was added for 4 hours of incubation in a CO_2_ incubator. Then, 100 μl of the culture medium was taken from each well and the absorbance (OD value) was measured using a microplate reader to determine cell viability. Cell viability was calculated using the formula: Cell viability = (sample OD450 nm / control OD450 nm) × 100%, cell inhibition rate = (1 - experimental group OD / control group OD) × 100%. Cell survival rate of the control group was set as 100%. Data represents the average of six parallel measurements.”

**Cell proliferation experiments:** The fibers were washed with deionized water until neutral and subsequently immersed in PBS buffer to remove moisture from the fibers. Next, the fibers soaked in PBS were subjected to high-pressure sterilization at 121°C for 20 minutes. The sterilized BC/OxBC fibers and (oxBC/BC)@PDA fibers were individually immersed in endothelial and smooth muscle culture media to replace the PBS present in the fibers. The fibers were then placed in a 48-well plate, and endothelial cells and smooth muscle cells were seeded on the fiber-containing wells at a density of 9×10^4^ cells per square centimeter. Following 1-7 days of cell culture, the culture medium in the wells was removed, and the cells on the fibers were stained for viability using a Calcein-AM/PI dual-staining kit.

**Cell Immunofluorescent Staining:** To further identify and characterize HUVES and HASMC on the material surface, immunohistochemical staining was performed. The cellular regions of the material were washed with PBS and fixed at room temperature for 20 minutes using 4% paraformaldehyde. Subsequently, the material was permeabilized at room temperature for 10 minutes using 0.1% Triton X-100, followed by washing with PBS. The material was then fully covered with a 3% BSA PBST (0.1% Tween) solution and incubated at room temperature for 1 hour in a humid chamber. The blocked material was incubated overnight at 4℃ with appropriate dilutions of primary antibodies: CD31 mouse monoclonal antibody (1:400, Proteintech) and α-SMA rabbit polyclonal antibody (1:100, Smooth muscle actin). The next day, the samples were washed with PBST (0.1% Tween) and incubated with fluorescently labeled diluted secondary antibodies: CyTM2-conjugated AffiniPure Donkey Anti-Mouse IgG (H+L) and CoraLite594-conjugated Goat Anti-Rabbit IgG (H+L), at room temperature for 1 hour. After incubation, the samples were washed again with PBST (0.1% Tween). DAPI-PBS (10 μg/ml) was added to the washed samples and incubated for 8 minutes. The samples were then rinsed with PBS and immersed in glycerol for confocal fluorescent microscopy scanning.

**Enzymolysis experiment of (oxBC/BC)@PDA hollow fibers:** Firstly, the (oxBC/BC)@PDA hollow fibers were washed with deionized water until they were neutral. Then, these fibers were put into an autoclave at 121 ℃ for sterilization treatment for 20 minutes. These fibers were evenly divided into 5 samples with the same weight, and soaked in the mixed solution of neutral cellulase, β-Glucosidase and PBS in petri dishes. The concentrations of Neutral cellulase and β-glucosidase were 4 mg/ml and 6 mg/ml, respectively. These petri dishes were placed in the cell incubator. With the enzymatic hydrolysis of BC, these fibers will continuously peel off some fragments. After a certain number of days (1, 3, 7, 14, 21 days), a petri dish was taken out for photos, and weigh the dry weight of the samples and fragments. All the treatment processes were carried out in a sterile environment.

Record the mass data of the remaining fibers at each time point and calculate the mass loss rate of the fibers at different times. Let the initial fiber mass be *m_0_* and the remaining fiber mass at a certain time point be *m_t_*, then the mass loss rate:

$X=\frac{m_{0}-m_{t}}{m_{0}}$ (s1)

According to the first-order kinetic equation:

$\ln\frac{1}{1-X}=kt$ (s2)

where *k* is the degradation rate constant and *t* is the reaction time), perform linear regression fitting on the obtained data. Determine the degradation rate constant through the slope of the fitting line, so as to deeply analyze the degradation kinetic characteristics of SO-(oxBC/BC)@PDA hollow fibers under the action of cellulase.

**Permeability experiment of (oxBC/BC)@PDA hollow fiber:** Firstly, three (oxBC/BC)@PDA hollow fibers with a length of 10 cm were washed with deionized water until they were neutral. The water in the hollow cavities of these hollow fibers was drained by injecting air with a syringe. Then, fluorescent labeled dextran solutions with molecular weights of 20 kDa, 70 kDa and 2000 kDa was injected into the cavities of these three (oxBC/BC)@PDA hollow fibers respectively. Subsequently, the three (oxBC/BC)@PDA hollow fibers were sealed at both ends with wax and immersed in water. Finally, the fluorescent photos of these fibers were taken at a specific time (0 hour, 1 hour and 2 hours).

**Cell culture on the inner and outer surfaces of hollow fibers:** To demonstrate the growth of HUVES and HASMC on the hollow fibers, we first traced the two types of cells, with HUVES labeled in green (CMFDA, Invitrogen) and HASMC labeled in red (CMTPX, Invitrogen). The BC/OxBC@PDA hollow fiber was placed into a 6-well plate, and HASMC with red fluorescence were seeded onto the material in the well at a cell density of 5×10^4^/cm^2^. After two days of cultivation, the material was flipped, and HASMC with red fluorescence were seeded onto the material again at a cell density of 9×10^4^/cm^2^. Two more days of cultivation followed. The fibers, with HASMC adhering to their outer surfaces, were suspended in a U-shaped trough. The hollow fiber lumen was infused with HUVES suspension that was tagged green, and the cell density was 5×10^5^/ml. After two days of cultivation, the fiber was flipped, and HUVES tagged green was infused again at a cell density of 5×10^5^/ml. The fiber with cells was then washed using PBS, fixed with 4% paraformaldehyde for 30 minutes, washed again, sliced with an scissor, and kept moist with glycerol. The sample was then scanned with confocal fluorescence microscopy.

**Material implantation surgery:** Animals were randomly assigned to the control, sham or implant group. Implant for material implantation group, sham for implant control group, and control for blank control group. Before implantation, animals were anaesthetized with 4% isoflurane and maintained with 2% isoflurane in oxygen, using an Anesthesia Machine (RWD Life Science Co., Shenzhen, China). After hair shaving and skin disinfection of the right hind limb, cut an opening about 8 mm long on the surface of the skin and separated the flesh bluntly. Prepared the 8 mm material with an inner diameter of 1.5 mm and an outer diameter of 2.2 mm, and washed to remove excess solvent, implant the material subcutaneously in mice after sterilization.

The incisions were then closed and sterilized. The animals were ventilated with 100% oxygen until autonomous breathing was recovered. 7, 14, 28 days after the implantation, the animals were killed by CO2 inhalation to evaluate the attachment, immune response, fibrosis of the implant. The tissue of interest was excised and fixed in 4% paraformaldehyde solution for histological analysis.

**Immunofluorescence staining and image analyses:** The tissues of interest were excised and fixed by 4% paraformaldehyde solution for paraffin-slides. Transverse sections in 3 μm thickness for the tissues of interest were collected for immunofluorescent staining. We use the TSAPLus fluorescent double staining kit (Servicebio, Wuhan, Hubei, China), which has higher sensitivity. Macrophage M2 polarization was measured using CD206 and macrophage M1 polarization was measured using CD86.

Primary antibodies were used: Rabbit Anti-CD206 (1:3000, Servicebio, GB113497, Wuhan, Hubei, China), Rabbit Anti-CD86 (1:200, Servicebio,GB13585, Wuhan, Hubei, China). Secondary antibody were used correspondingly: HRP-conjugated Goat Anti-Rabbit (1:500, Servicebio, GB23303), CY3-conjugated Goat Anti-Rabbit (1:300, Servicebio, GB21303).

Fluorescence images were photographed by Nikon Eclipse C1 fluorescence microscopy. Scan tissue sections using the Nikon DS-U3 imaging system. Images were analyzed by an investigator blinded to experimental groups with ImageJ software (NIH, Bethesda, MD,USA).

Tissues paraffin sections were obtained for hematoxylin and eosin (H&E) or Masson staining. Tissue sections were scanned by LEICA-Aperio CS2 pathological slide scanner.

**Quantitative Real-time PCR:** Cells prepared from the tissues of interest were subjected to RNA extraction followed by cDNA synthesis. Expression of CCL5, CCL2, ICAM-1, TNF-α were detected by Real-time PCR. The primers used were CCL5: sense 5’-TGCTCCAATCTTGCAGTCGT-3’ and antisense 5’- GCAAGCAATGACAGGGAAGC-3’; ICAM-1: sense 5’- GTACTGTACCACTCTCAAAATAACTGG-3’ and antisense 5’- TGGGGCTTGTCCCTTGAGT-3’; CCL2: sense 5’- CAGGTCCCTGTCATGCTTCT-3’ and antisense 5’- GTGGGGCGTTAACTGCATCT-3’; TNF-α: sense 5’-CCCTCACACTCACAAACCACC-3’ and antisense 5’-CTTTGAGATCCATGCCGTTG-3’.

**ELISA:** The tissue was prepared into a 10% homogenate, and the supernatants was determined by ELISA. ELISA plates were coated with capture antibodies against mouse CCL5 (EK2129/2, MULTI SCIENCES,China)、CCL2 (EK287/2, MULTI SCIENCES,China)、ICAM-1 (CSB-E04575m, CUSABIO,China)、TNF-α (88-7324, Invitrogen,USA) . Non-specific binding was blocked by adding 200μl of blocking buffer per well for 2 h at room temperature. Biotinylated detection antibodies were added to the plates and incubated for 1 h at room temperature, then TMB substrate solution was added for 10-30 min at room temperature in the dark. The reaction was stopped with 100 μl 2M sulfuric acid. Finally, use a microplate reader (INFINITE 200 PRO, TECAN, Switzerland) to measure the OD value of each well at 450 nm to calculate the sample concentration.

**Calculating formulas and derived process of the spiral angle of the SO-oxBC/BC fiber (*θ_1_*)**

The orientation of oxBC nanofibrils results from the combined effects of axial shear during the extrusion process and circumferential shear from the rotating coagulation bath. The spinning needle is positioned at a specific distance from the center axis of the rotary coagulation bath, and the spinning fluid is injected into the bath through fine pipes and needles. The flow shear effect of spinning fluid in the channels of fine pipes and needles will tend to align the one-dimensional oxBC nanofibrils inside along the axial direction. During the spinning process, the coagulation bath rotates continuously at a defined speed to generate a circumferential shear flow field. When the spinning fluid is injected into the coagulation bath, its free end, distant from the spinning needle, rotates under the influence of the circumferential shear flow field. The rotational speed of the spinning fluid (*ω_spinning-fluid_*) matches the angular velocity of the coagulation bath, such that *ω_spinning-fluid_* = *ω_coagulation bath_*. However, the end of the spinning fluid near the needle remains stationary. This differential in rotational movement causes the fluid to twist, reorienting the oxBC nanofibrils from an axial to a spiral alignment. This twisting mechanism is analogous to the process of yarn twisting, suggesting that the spiral angle of the spinning fluid (*θ_0_*) conforms to the yarn twisting angle formula:

$$\tan\theta_{0}=\frac{C_{0}}{\Delta l_{0}}=\frac{\pi\times d_{0}}{\Delta l_{0}}=\frac{2\times\pi\times\sqrt{\frac{S_{0}}{\pi}}}{\Delta l_{0}} (S2)$$

Where *C_0_* represents the circumference of the spinning fluid cross-section, *Δl_0_* the spiral pitch of the spinning fluid, *d_0_* the diameter of the spinning fluid cross-section, and *S_0_* the cross-sectional area of the spinning fluid.

The coagulation bath promotes the solidification of the spinning fluid into primary fibers, which are then subjected to partial solvent removal to yield SO-oxBC/BC fibers. During this process, the fibers experience significant, uneven volume shrinkage in both axial and circumferential directions. The diameter and spiral pitch of the fibers after shrinkage are denoted as *d_1_* and *Δl_1_*​, respectively, with the shrinkage ratios in the circumferential and axial directions represented by *a* and *b*, respectively. Thus, the spiral angle of the SO-oxBC/BC fiber (*θ_1_*) can be described by the following equation:

$$\tan\theta_{1}=\frac{C_{1}}{\Delta l_{1}}=\frac{a\times\pi\times d_{0}}{b\times\Delta l_{0}}=\frac{a}{b}\times\frac{2\times\pi\times\sqrt{\frac{S_{0}}{\pi}}}{\Delta l_{0}} (S3)$$

Where *C_1_* represents the circumference of oxBC/BC fiber cross-section.

According to classical flow rate and velocity equations, *Δl_0_* and *S_0_* follow the relations:

$$\Delta l_{0}=v_{fiber}\times t=v_{fiber}\times\frac{2\times\pi}{\omega_{fiber}}=2\times\pi\times R (S4)$$

$$S_{0}=\frac{Q}{v_{fiber}}=\frac{Q}{\omega_{fiber}\times R}=\frac{Q}{\omega_{coagulation bath}\times R} \left( S5 \right)$$

Where *Q* represents the flow rate of the spinning fluid, and *v_fiber_* represents the rate of fiber length growth, which is equal to the linear velocity of fiber movement.

Substituting Equation S3 and S4 into Equation S2, we derive:

$$\tan\theta_{1}=\frac{a}{b}\times\frac{2\times\pi\times\sqrt{\frac{Q}{\pi\times\omega_{coagulation bath}\times R}}}{2\times\pi\times R}=\frac{a}{b}\times\sqrt{\frac{Q}{\pi\times\omega_{coagulation bath}\times R^{3}}} (S6)$$

These equations indicate that nanofibril orientation is positively correlated with *Q* and inversely correlated with *ω_coagulation bath_* and *R*, consistent with experimental observations.


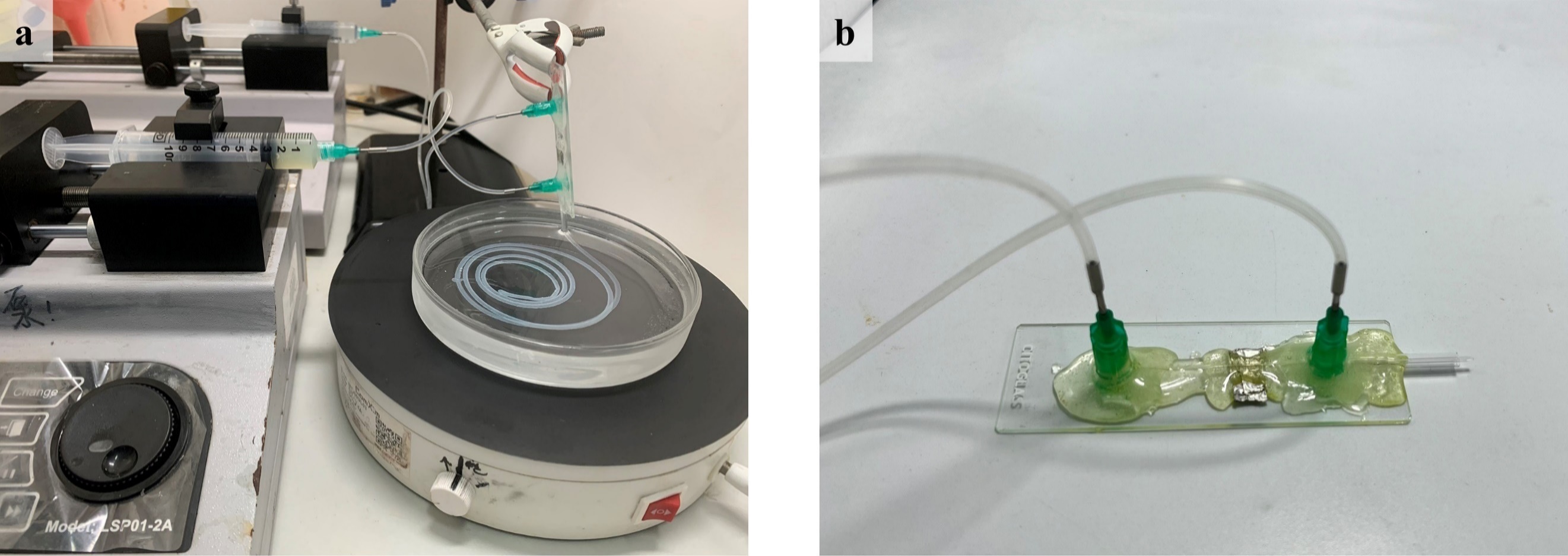


**Figure S1** Optical photos of microfluidic coaxial spinning device. (a) the whole spinning system; (b) the coaxial microfluidic chip.


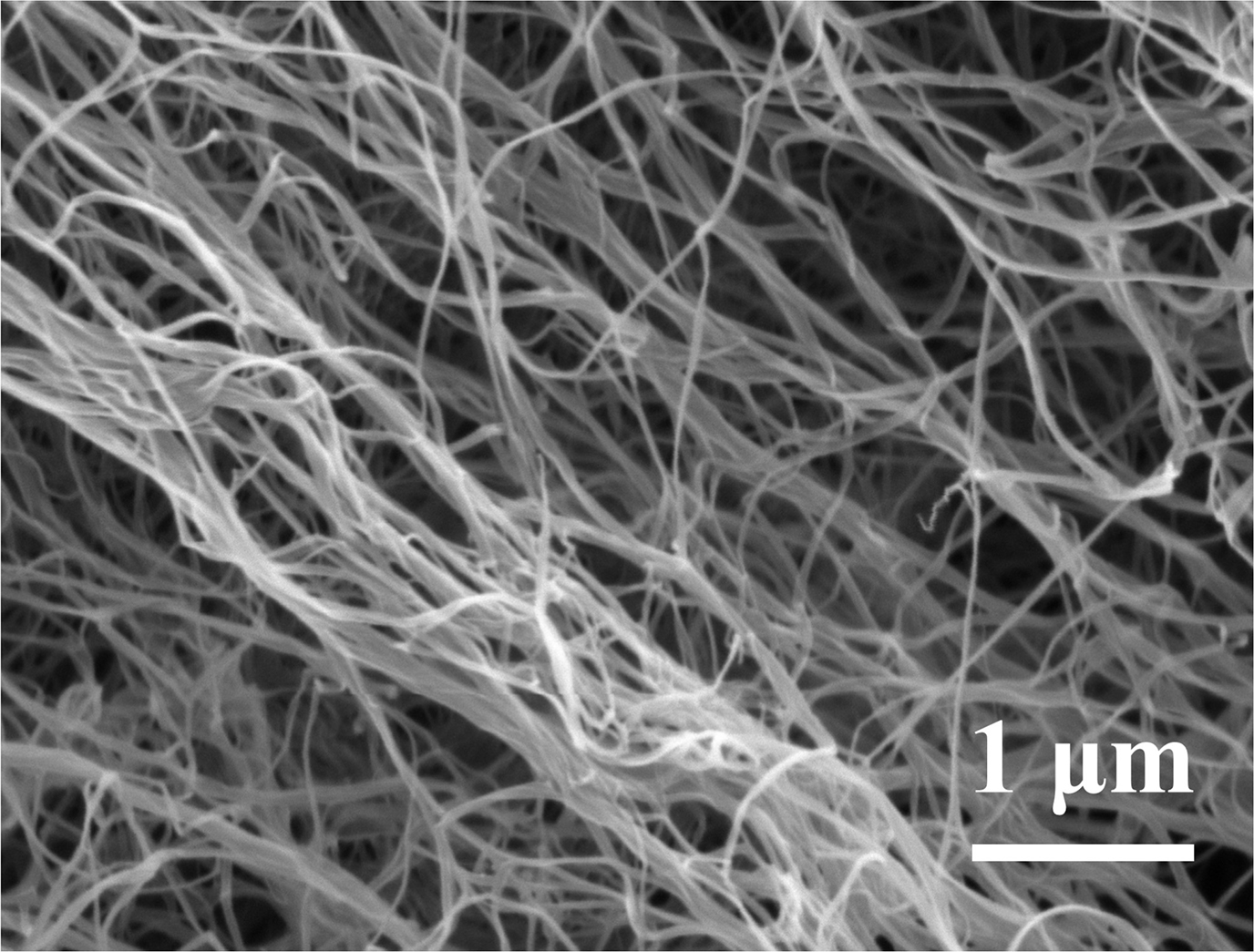


**Figure S2** SEM image of the BC. Scale bar: 1 μm.


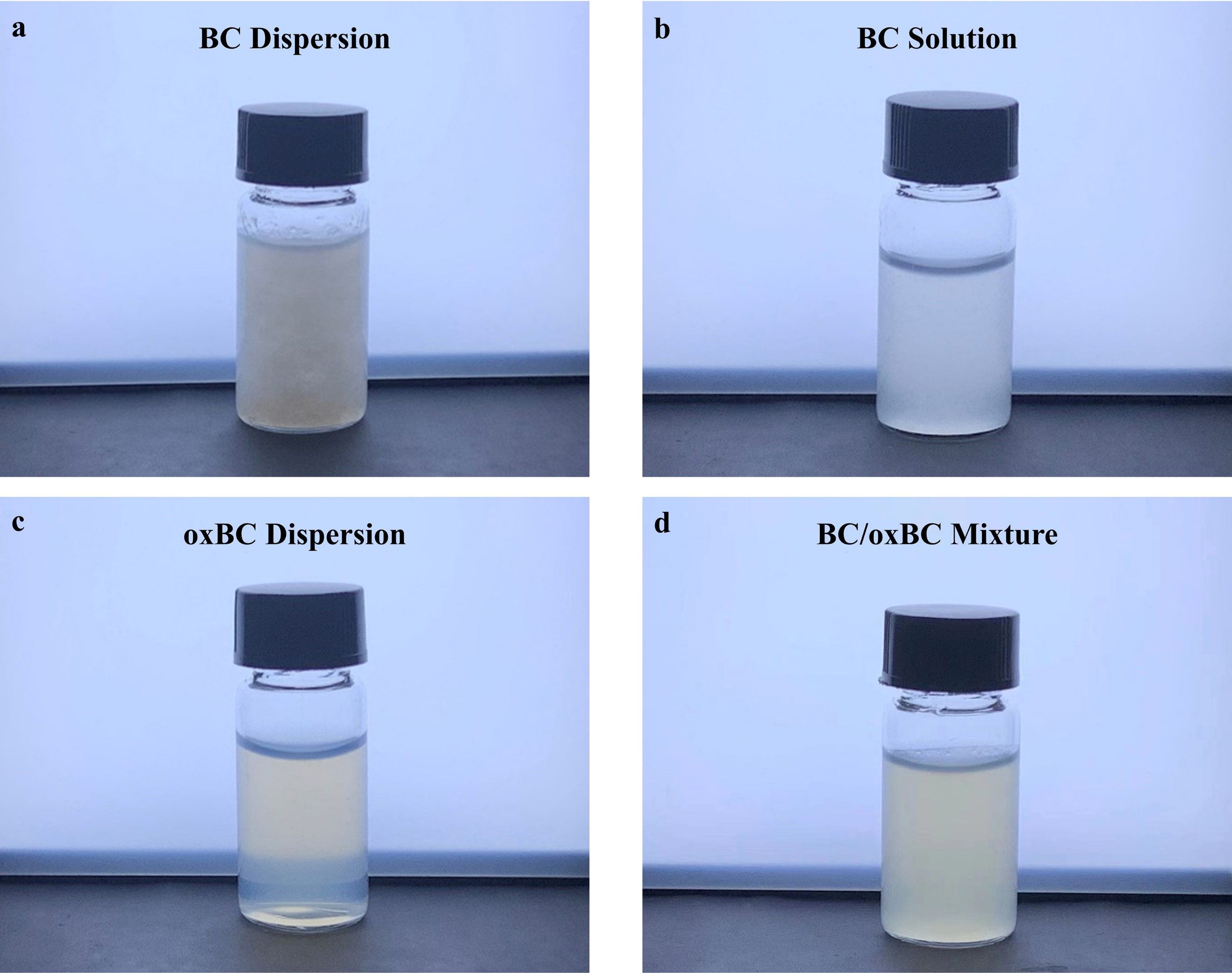


**Figure S3** Optical photos of various BC based fluids. (a) BC dispersion; (b) BC solution; (c) oxBC dispersion; (d) oxBC/BC mixture.


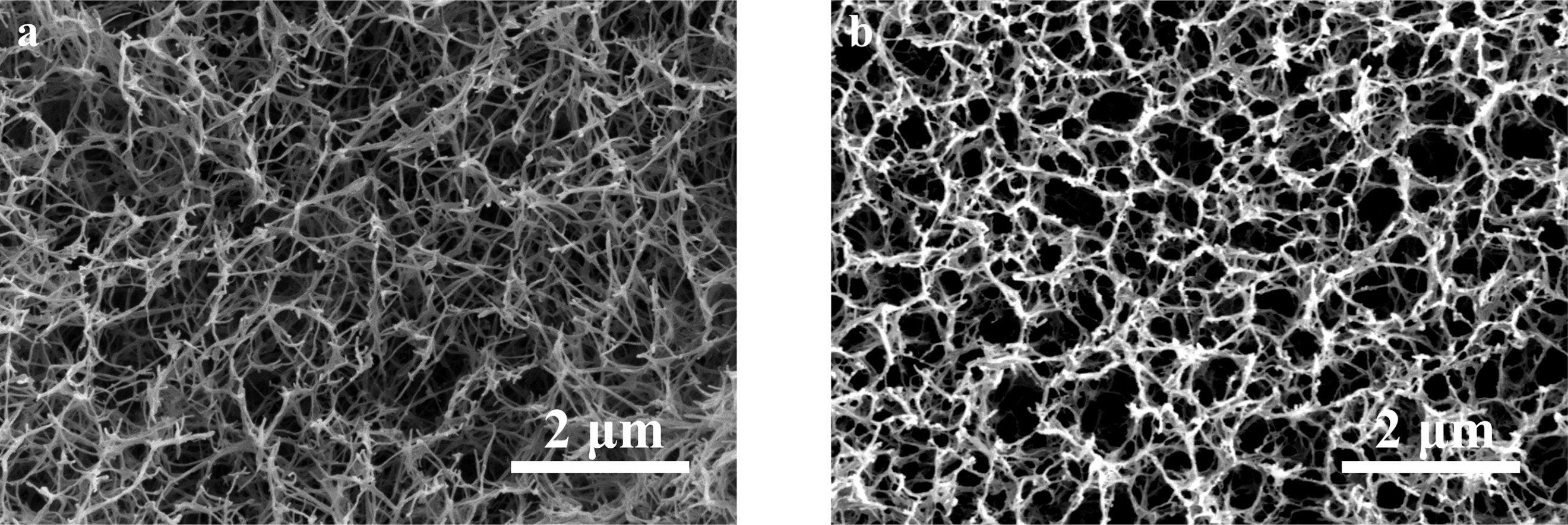


**Figure S4** SEM cross-section morphology of neat oxBC fiber (a) and SO-oxBC/BC fiber (b).


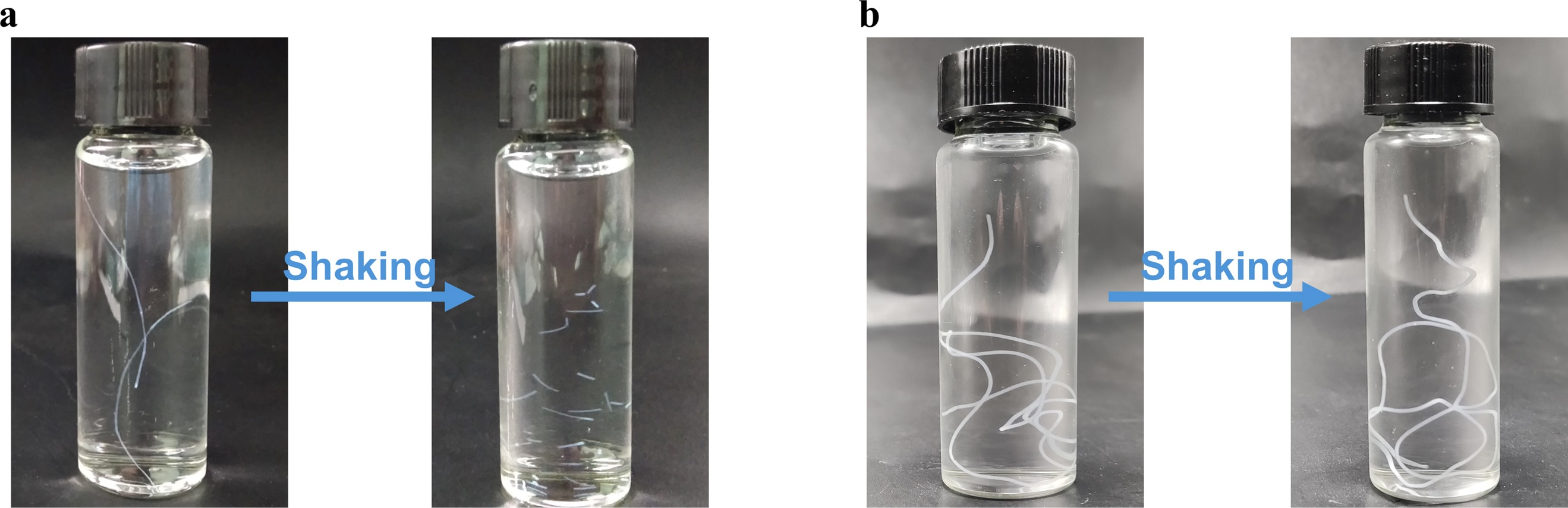


**Figure S5** Optical photos of neat oxBC fiber (a) and SO-oxBC/BC fiber (b) before and after soaking and shaking in water.


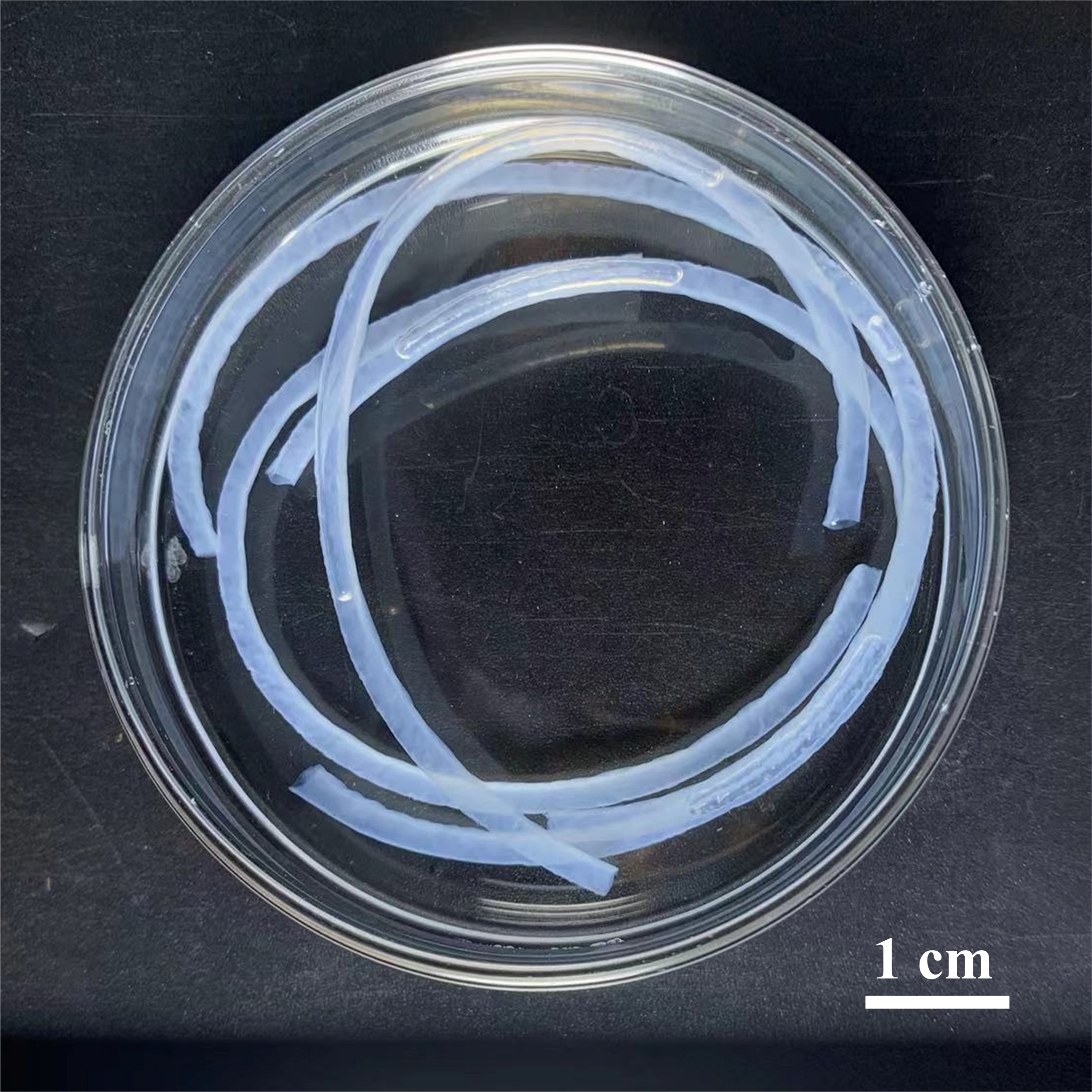


**Figure S6** Optical photo of SO-oxBC/BC fibers. Scale bar: 1 cm.


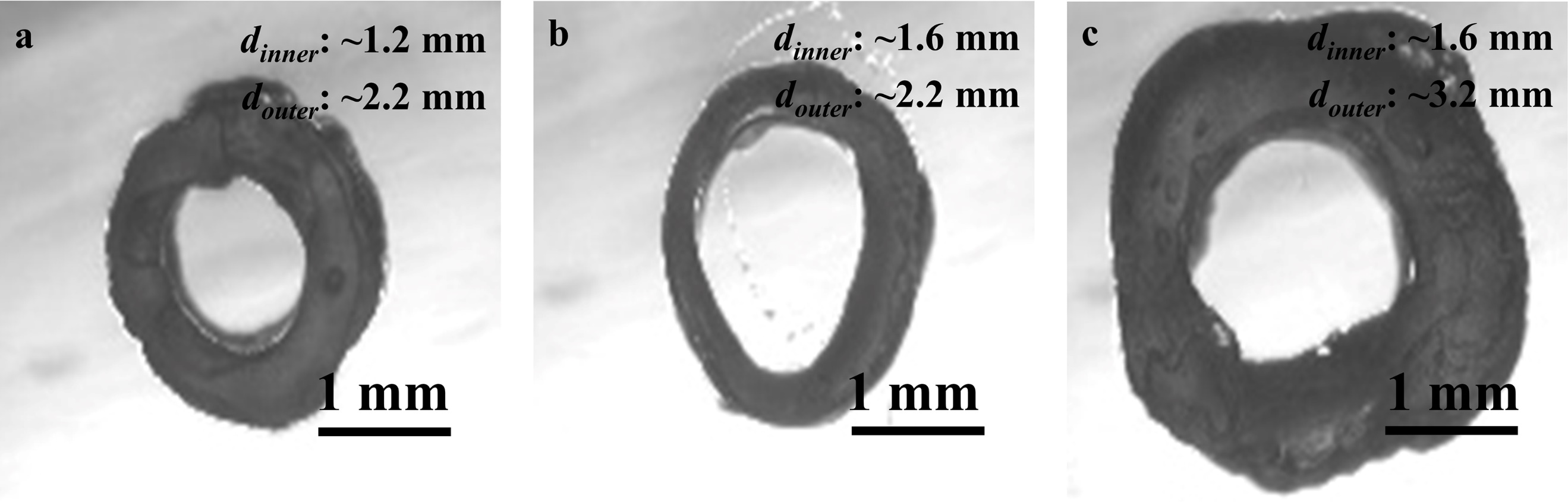


**Figure S7** Microscopic photos of SO-oxBC/BC fibers with different inner and outer diameters prepared using microfluidic coaxial devices with different geometric parameters. Scale bar: 1 mm


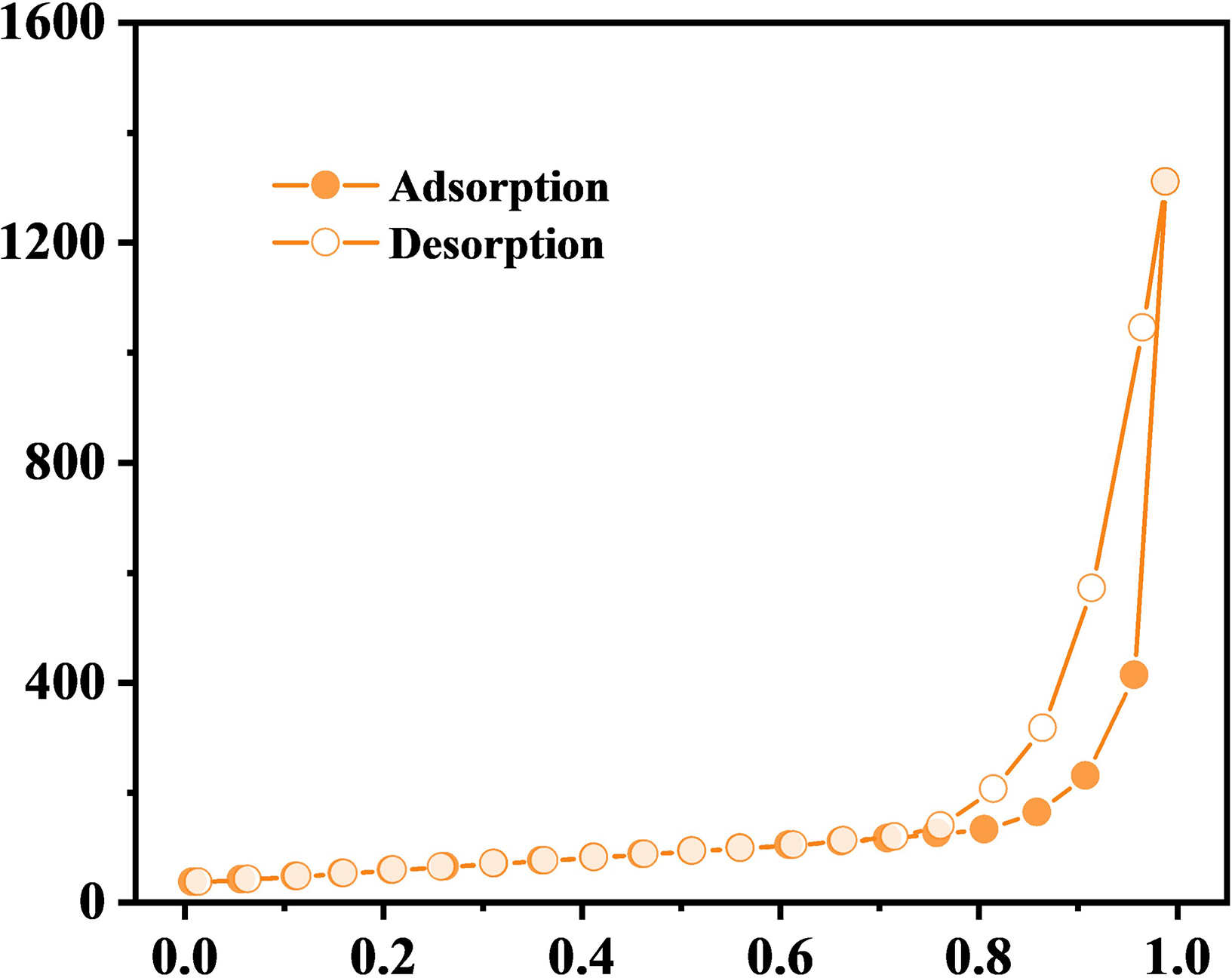


**Figure S8** N_2_ adsorption/desorption isotherms of SO-oxBC/BC fibers.


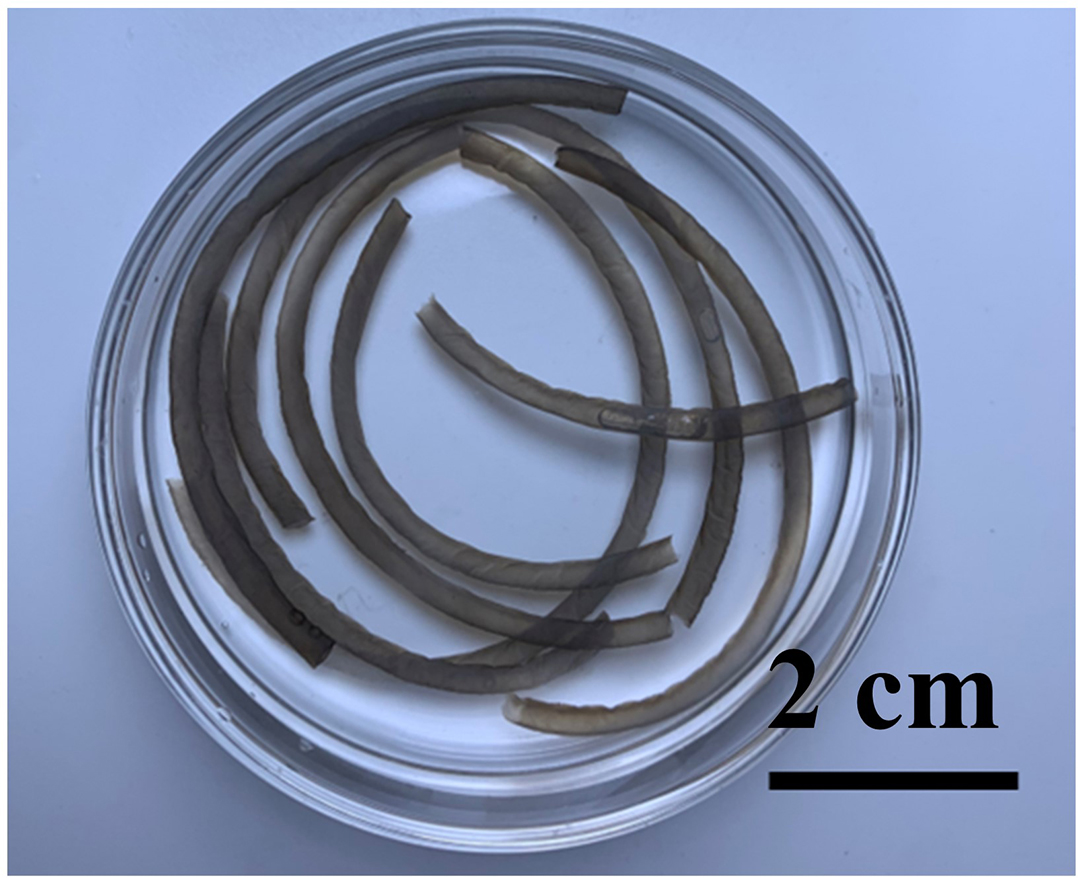


**Figure S9** Optical photo of SO-(oxBC/BC)@PDA hollow fiber. Scale bar: 2 cm.


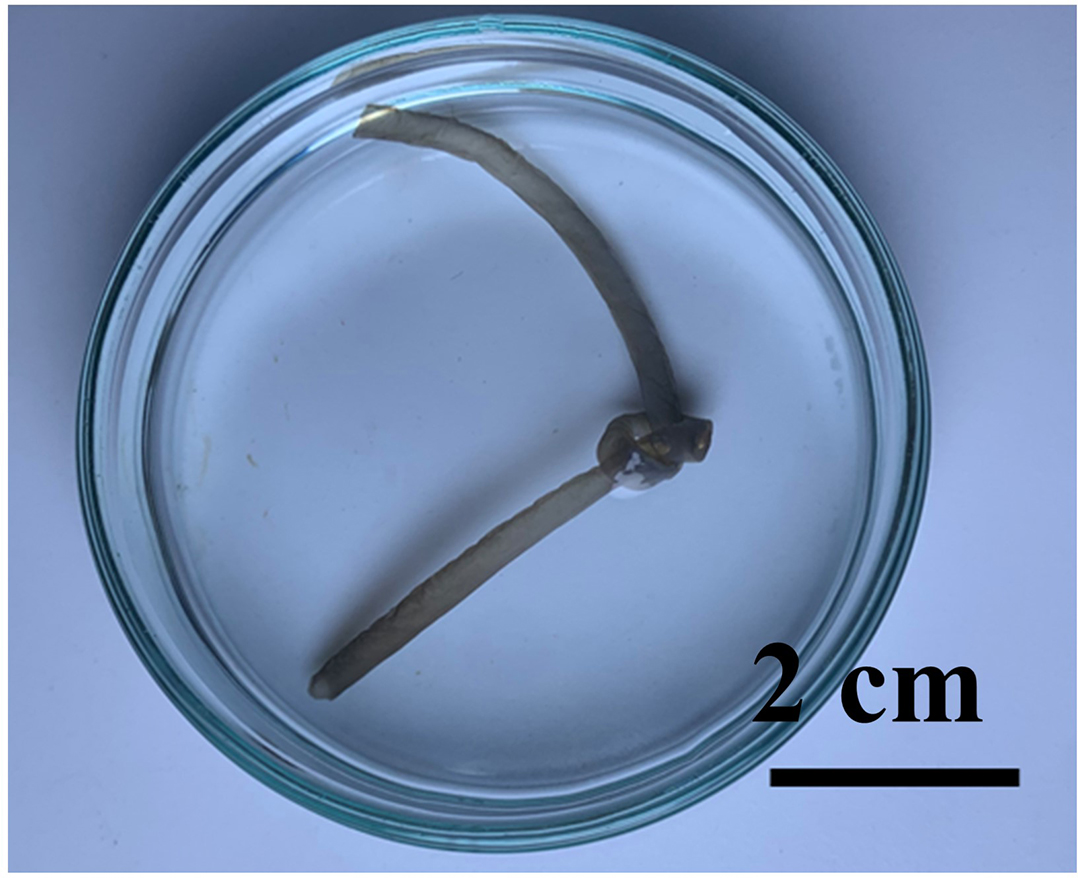


**Figure S10** Optical photo of knotted SO-(oxBC/BC)@PDA hollow fiber. Scale bar: 2 cm.


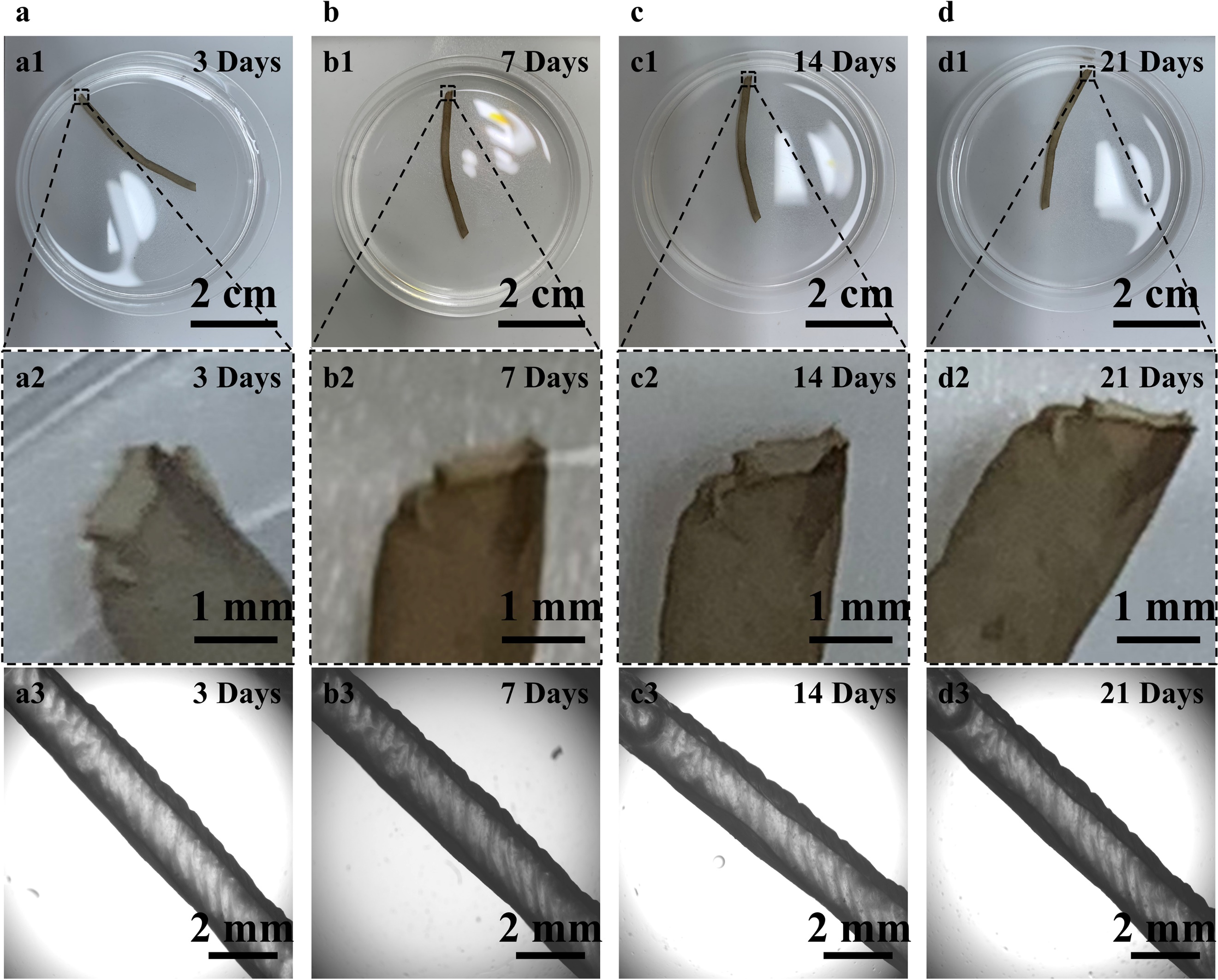


**Figure S11** Optical and microscopic photos of SO-(oxBC/BC)@PDA fibers soaked in water for specific days. (a1, b1, c1, d1) optical photos of SO-(oxBC/BC)@PDA fibers soaked in water for 3, 7, 14 and 21 days respectively. Scale bar: 2 cm; (a2, b2, c2, d2) partial enlarged view of Figure a1, b1, c1 and d1 respectively. Scale bar: 1 mm; (a3, b3, c3, d3) microscopic photos of SO-(oxBC/BC)@PDA fibers soaked in water for 3, 7, 14 and 21 days respectively. Scale bar: 2 mm.


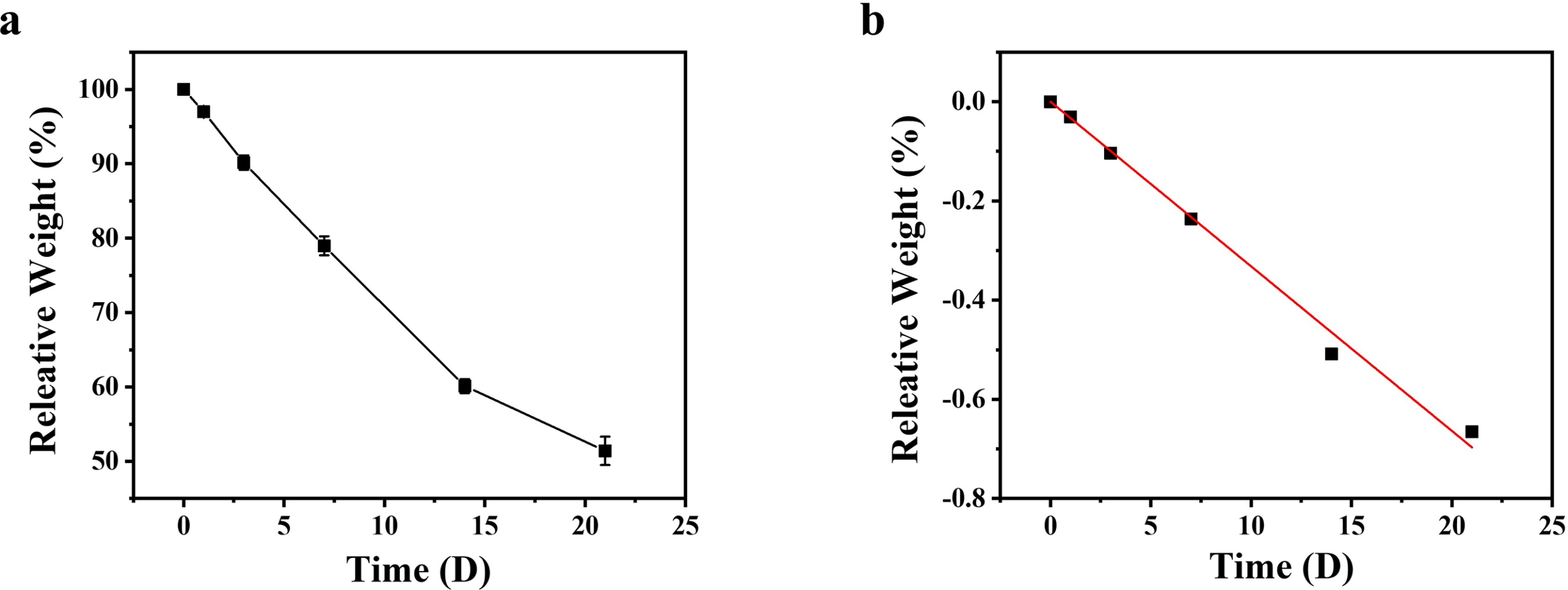


**Figure S12** The weight loss curve (a) and first-order kinetic fitting diagram (b) of SO-(oxBC/BC)@PDA fibers under the action of neutral cellulase and β – glucosidase.


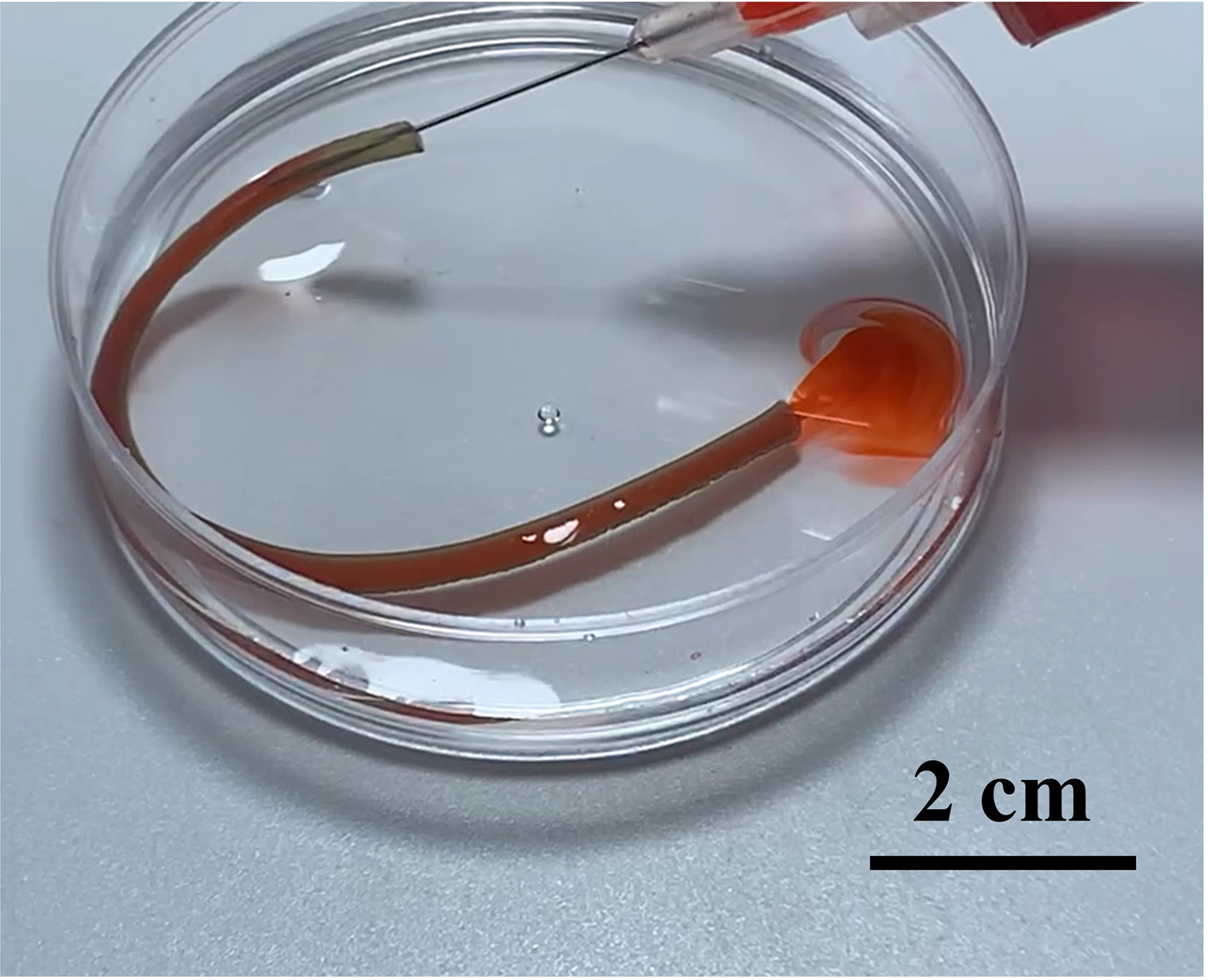


**Figure S13** Optical photo of SO-(oxBC/BC)@PDA hollow fiber perfused with red dye solution. Scale bar: 2 cm.


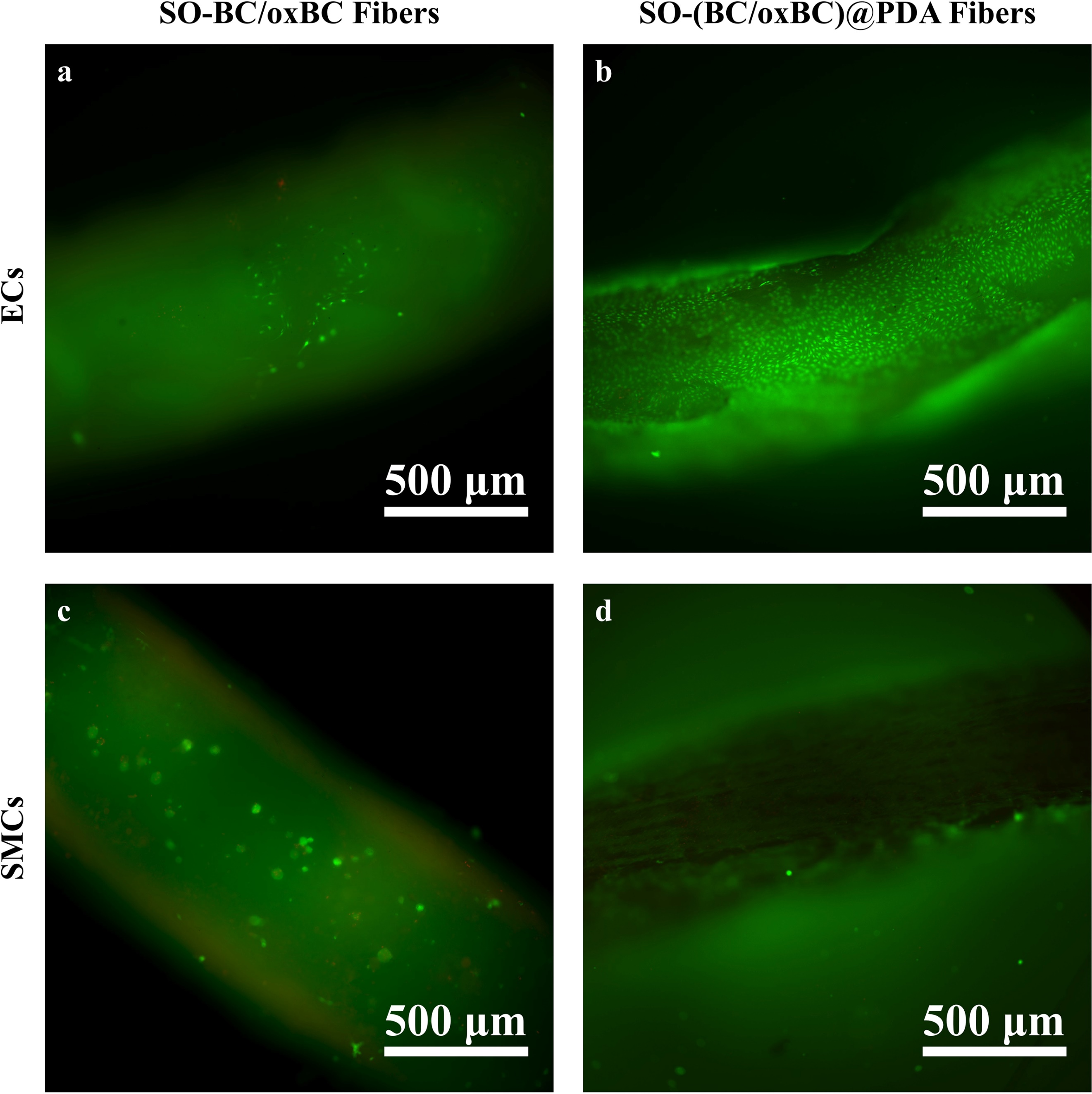


**Figure S14** Fluorescence photos of ECs and SMCs adhering to the surfaces of SO-oxBC/BC fibers and SO-(oxBC/BC)@PDA fibers. (a) ECs adhering to the surfaces of SO-oxBC/BC fibers; (b) ECs adhering to the surfaces of SO-(oxBC/BC)@PDA fibers; (c) SMCs adhering to the surfaces of SO-oxBC/BC fibers; (d) SMCs adhering to the surfaces of SO-(oxBC/BC)@PDA fibers. Scale bar: 500 μm.


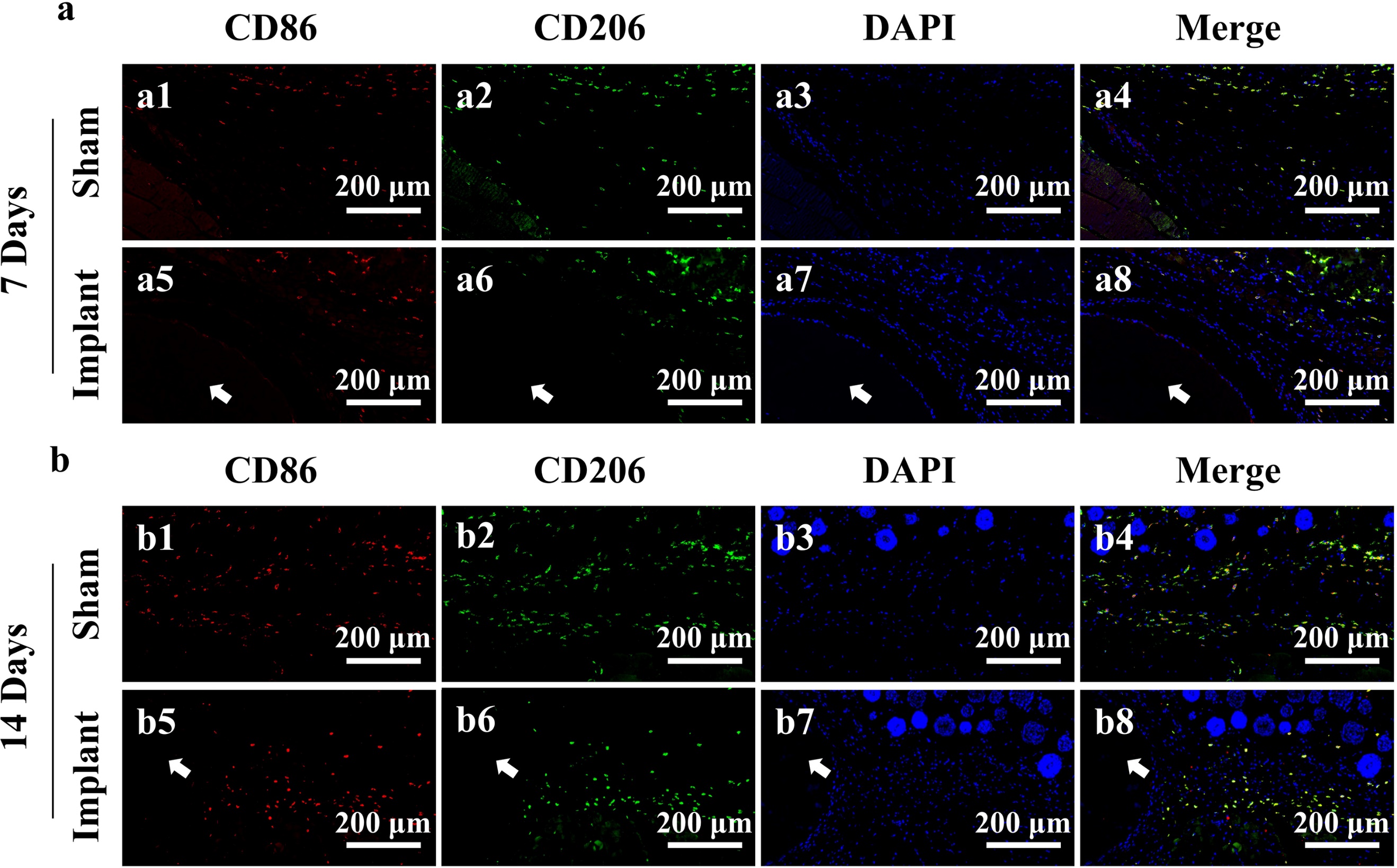


**Figure S15** Fluorescence photos of macrophage at the implantation site of mice after 28 after specific days of SO-(oxBC/BC)@PDA fibers implantation. (a) 7 days; (b)14 days. Scale bar: 200 μm.


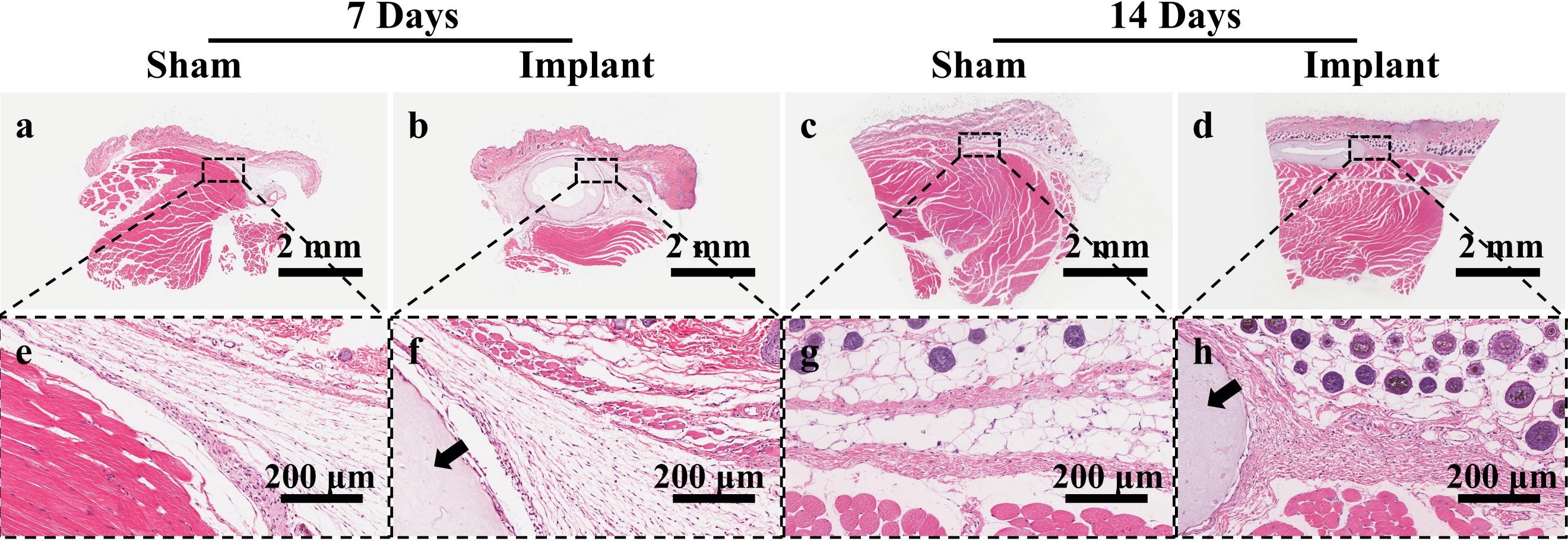


**Figure S16** Microscopic images and partial enlarged view of HE-stained tissue slices of mice after specific days (7 days, 14days) of SO-(oxBC/BC)@PDA fibers implantation. (a, e) sham group, 7 days; (b, f) implant group, 7days; (e, g) sham group, 14 days; (d, h) implant group, 14days. The arrows indicate the position of the SO-(oxBC/BC)@PDA fibers. Scale bar: 2 mm (a-d) and 200 μm (e-h).


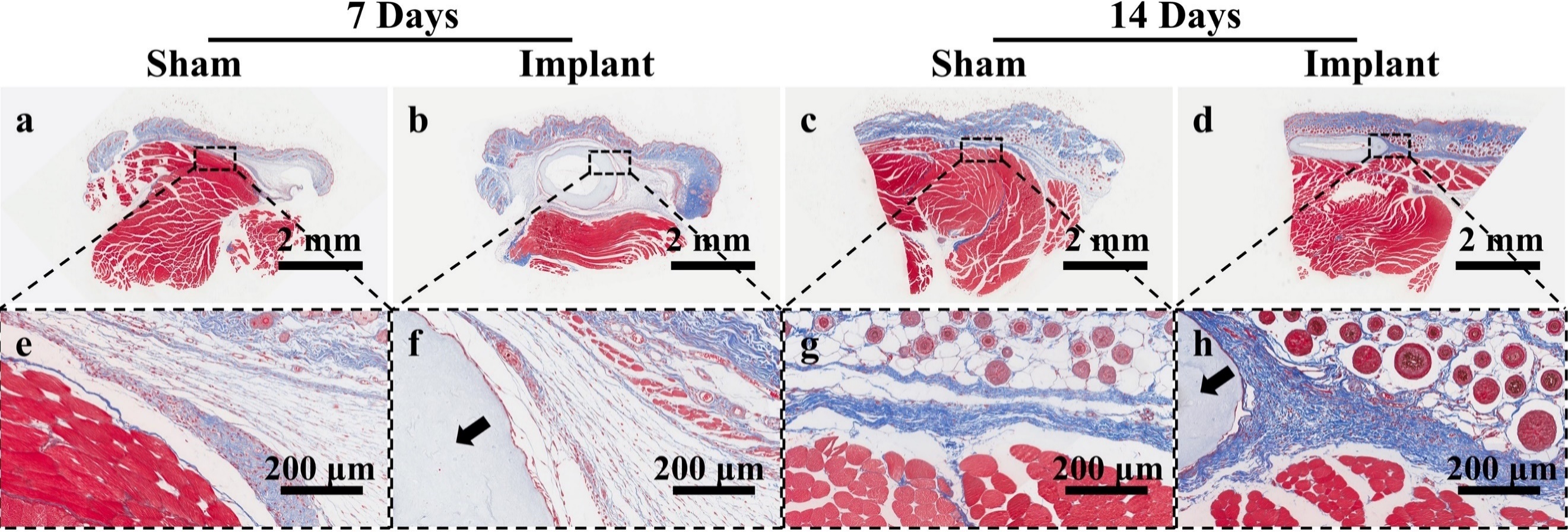


**Figure S17** Microscopic images and partial enlarged view of Masson-stained tissue slices of mice after specific days (7 days, 14days) of SO-(oxBC/BC)@PDA fibers implantation. (a, e) sham group, 7 days; (b, f) implant group, 7days; (e, g) sham group, 14 days; (b, f) implant group, 14days. The arrows indicate the position of the SO-(oxBC/BC)@PDA fibers. Scale bar: 2 mm (a-d) and 200 μm (e-h).

**Table S1** Comparison table of various processing methods for constructing tubular tissue engineering scaffolds

| **Parameter** | **Microfluidic Spinning Based on Nanomaterial Dispersion (This Work)** | **3D Printing** | **Electrospinning** | **Laser Engraving** |
| --- | --- | --- | --- | --- |
| **Resolution** | Tens of nanometers (dependent on the size of nanomaterials). | Typically 20–200 µm | Fiber diameters can reach sub-micron scale. | Processing accuracy can reach sub-micron or even nanometer scale, but there are requirements for the high-temperature dimensional stability of the substrate. |
| **Scalability** | - Can be scaled by parallelizing microfluidic channels.  - Produces continuous fibers at relatively high throughput. | - Print sizes are limited by printer build volume and time.  - Industrial-scale printers offer larger volumes but can be costly and slower for large builds. | - Relatively high throughput is possible, but requires careful solvent handling and collection of fibers.  - Typically collected as randomly oriented fibrous mats. | - The processing speed is greatly affected by the processing accuracy.  - Generally only suitable for processing on flat substrates. |
| **Structural / Self-Support** | High modulus nanomaterials can be used as the skeleton of the material to enhance mechanical stability. | Final printed structures can be mechanically robust, but layer adhesion may be a concern depending on material and method. | Fibrous mats generally lack self-supporting structure unless post-processed. | - Dependent on the substrate. |
